# Functional Characterization of Circulating Tumor Cells (CTCs) from Metastatic ER+/HER2− Breast Cancer Reveals Dependence on HER2 and FOXM1 for Endocrine Therapy Resistance and Tumor Cell Survival: Implications for Treatment of ER+/HER2− Breast Cancer

**DOI:** 10.3390/cancers13081810

**Published:** 2021-04-10

**Authors:** Sven Roßwag, Cristina L. Cotarelo, Klaus Pantel, Sabine Riethdorf, Jonathan P. Sleeman, Marcus Schmidt, Sonja Thaler

**Affiliations:** 1 European Center for Angioscience, Medical Faculty Mannheim, University of Heidelberg, 68167 Mannheim, Germany or Sven.Rosswag@web.de (S.R.); Jonathan.Sleeman@medma.uni-heidelberg.de (J.P.S.); 2 Institute of Pathology, University Medical Center of Heinrich-Heine University, 40225 Duesseldorf, Germany; Cristina.Cotarelo@med.uni-duesseldorf.de; 3 Institute of Tumor Biology, University Medical Center Hamburg-Eppendorf, 20246 Hamburg, Germany; pantel@uke.de (K.P.); s.riethdorf@uke.de (S.R.); 4 Karlsruhe Institute of Technology (KIT) Campus Nord, Institute of Biological and Chemical Systems—Biological Information Processing, 76344 Eggenstein-Leupoldshafen, Germany; 5 Department of Gynecology and Obstetrics, University Medical Center of Johannes Gutenberg University, 55131 Mainz, Germany; Marcus.Schmidt@unimedizin-mainz.de

**Keywords:** ER+/HER2− circulating tumor cells, endocrine therapy resistance, HER2-dependent FOXM1 expression

## Abstract

**Simple Summary:**

Acquired endocrine resistance and late recurrence in patients with ER+/HER2− breast cancer are complex and not fully understood. Here, we evaluated mechanisms of acquired resistance in circulating tumor cells (CTCs) from an ER+/HER2− breast cancer patient who initially responded but later progressed under endocrine treatment. We found a switch from ERα-dependent to HER2-dependent and ERα-independent expression of FOXM1, which may enable disseminated ER+/HER2− cells to re-initiate tumor cell growth and metastasis formation in the presence of endocrine treatment. We found that NFkB signaling sustains HER2 and FOXM1 expression in CTCs in the presence of ERα inhibitors suggesting that NFkB and FOXM1 might be an efficient therapeutic approach to prevent late recurrence and to treat endocrine resistance. Collectively our data show that CTCs from patients with endocrine resistance allow mechanisms of acquired endocrine resistance to be delineated, and can be used to test potential drug regimens for combatting resistance.

**Abstract:**

Mechanisms of acquired endocrine resistance and late recurrence in patients with ER+/HER2− breast cancer are complex and not fully understood. Here, we evaluated mechanisms of acquired resistance in circulating tumor cells (CTCs) from an ER+/HER2− breast cancer patient who initially responded but later progressed under endocrine treatment. We found a switch from ERα-dependent to HER2-dependent and ERα-independent expression of FOXM1, which may enable disseminated ER+/HER2− cells to re-initiate tumor cell growth and metastasis formation in the presence of endocrine treatment. Our results also suggest a role for HER2 in resistance, even in ER+ breast cancer cells that have neither HER2 amplification nor activating HER2 mutations. We found that NFkB signaling sustains HER2 and FOXM1 expression in CTCs in the presence of ERα inhibitors. Inhibition of NFkB signaling blocked expression of HER2 and FOXM1 in the CTCs, and induced apoptosis. Thus, targeting of NFkB and FOXM1 might be an efficient therapeutic approach to prevent late recurrence and to treat endocrine resistance. Collectively our data show that CTCs from patients with endocrine resistance allow mechanisms of acquired endocrine resistance to be delineated, and can be used to test potential drug regimens for combatting resistance.

## 1. Introduction

Around 70% of breast cancers express the estrogen receptor alpha (ERα) and depend on estrogen for growth and disease progression. The targeting of ERα with tamoxifen or aromatase inhibitors (AIs) is therefore the standard treatment for ER+ breast cancer [1]. Although most ER-positive/HER2-negative (ER+/HER2−) breast cancers are associated with good prognosis, around one third of patients progress and develop late recurrences, sometimes even decades after initial diagnosis and treatment [2,3].

Endocrine therapy in patients with ERα+ breast cancer has significantly improved patient outcomes [4]. However, resistance towards endocrine therapies occurs often, and is found in nearly all hormone receptor positive breast cancer patients with metastatic disease [2,3].

The forkhead-box-protein M1 (FOXM1) is a transcriptional regulator whose expression is associated with resistance to endocrine therapies in ER+ breast cancers and metastatic progression [5,6]. FOXM1 fosters the outgrowth of ER+ breast cancer cells and circumvents drug-induced senescence in the presence of endocrine therapies [5]. FOXM1 also increases the size of the stem cell population, and promotes cell growth, invasiveness and metastasis [5,6]. Consistently, knockdown of FOXM1 in breast cancer cells can restore sensitivity to endocrine therapies [5]. Despite such insights into mechanisms of endocrine therapy resistance, and the development of new strategies for the treatment of endocrine resistant ER+/HER2− metastatic breast cancers such as the use of mTOR, CDK4/6 and PIK3CA inhibitors, the management of advanced hormone-receptor positive breast cancer with resistance to endocrine therapies as well as strategies to avoid late recurrence remain a significant challenge [7,8].

One obstacle to the investigation of mechanisms that lead to endocrine therapy resistance is the lack of authentic in vitro and in vivo models. Most functional studies have used established ER+ breast cancer cell lines such as MCF7 and T47D. However, authentic in vitro models derived from ER+/HER2− breast cancer patients with acquired endocrine resistance that can be used for functional studies are rare. Experimental investigations of endocrine resistant mechanisms are mainly based on comprehensive genetic analysis of patient material [9,10,11,12,13] or patient derived xenografts [14,15]. These studies have provided information about permanent changes in the genome of breast cancer cells that cause resistance to endocrine treatment, such as constitutively activating ERα mutations in patients after treatment with multiple lines of endocrine therapy [15,16,17], or constitutively activating mutations in the PI3K/Akt/mTOR signaling pathway [7]. However such studies do not address transient adaptation mechanisms that might enable ER+/HER2− breast cancer cells to sustain proliferation that exist only in the presence of ERα inhibition. 

Circulating tumor cells (CTCs) have the potential to serve as markers for monitoring disease progression, and might be useful for the design of personalized treatment strategies [18,19]. However, functional studies with CTCs are difficult due to the very low numbers of CTCs in the peripheral blood of cancer patients [20] among others due to the harsh conditions within the blood flow [21]. Adaptation of CTCs to anti-cancer therapies and survival in the blood flow may require properties that do not exist in established standard cell lines. Thus, although functional characterization of CTCs is a challenge, these cells represent valuable tools for investigating survival mechanisms, metastasis formation and drug resistance.

In the present study, we evaluated whether circulating tumor cells (CTCs) from patients with acquired endocrine resistance could serve as an authentic model to investigate mechanisms leading to an attenuated response to endocrine treatment. Therefore, we performed functional studies with the cell line CTC-ITB-01 established from CTCs that were originally isolated from a patient with ER+/HER2− metastatic breast cancer after the patient progressed after chemotherapy and under endocrine treatment [22]. Genomic analyses show high concordance between CTC-ITB-01 and CTCs that were originally present in the cancer patient at the time point of blood draw [22]. Functional investigation of endocrine resistance mechanisms in CTC-ITB-01 cells should therefore provide insights into authentic endocrine resistance mechanisms that are present within patients, and which do not exist in standard cell lines.

Here we report significant differences between CTC-ITB-01 cells and ER+/HER2− MCF7 cells with regard to susceptibility towards pharmacological ERα inhibition. Although CTC-ITB-01 cells do not harbor any ERα mutations [22], they displayed no significant response towards fulvestrant and AZD9496. Both CTC-ITB-01 and MCF7 cells show increased expression of HER2 upon endocrine treatment. However, in contrast to MCF7 cells, pharmacological ERα inhibition in CTC-ITB-01 cells did not decrease expression of FOXM1. HER2 was required to sustain FOXM1 expression, cell growth and survival in CTC-ITB-01 cells in the presence of ERα inhibitors. This suggests that there is a switch from ERα-dependent to HER2-dependent and ERα-independent expression of FOXM1 when patients progress under endocrine treatment, even in ER+ breast cancer cells that do not have HER2 amplification or activating HER2 mutations. Mechanistically, we found that HER2 and FOXM1 expression in CTC-ITB-01 cells depends on NFkB. Suppression of HER2 and FOXM1 and induction of cell death was strongly augmented when NFkB inhibition was combined with fulvestrant treatment, underscoring the role of NFkB signaling for sustained expression of HER2 and FOXM1 in the presence of ERα inhibition. We conclude that CTCs from patients with endocrine resistance are useful for functional investigations into mechanisms of acquired endocrine resistance, and allow the testing of potential drug regimens to target these mechanisms.

## 2. Results

### 2.1. CTC-ITB-01 Cells Established from a Patient with Metastatic ER+/HER2− Breast Cancer Show No Response to Pharmacological Inhibition of ER alpha

To investigate mechanisms that enable ER+/HER2− breast cancer CTCs to escape from ERα inhibition, we used CTCs from an ER+/HER2− breast cancer patient who initially responded but later progressed under endocrine treatment. CTCs were isolated from the blood of a patient after the patient progressed under endocrine therapy, and established in culture as a permanent cell line designated CTC-ITB-01 [22].

The patient was originally diagnosed with bi-lateral ER+/HER2− breast cancer (invasive ductal carcinoma (IDC) and invasive lobular carcinoma (ILC) [22] (Figure S1). First, we therefore used immunohistochemical (IHC) staining to determine whether the CTCs (hereafter referred to as CTC-ITB-01 cells) are derived from IDC or ILC cells. Furthermore, we also performed HER2 CISH to exclude any possible expansion of cells with ER+/HER2 amplification. 

**Figure 1:**
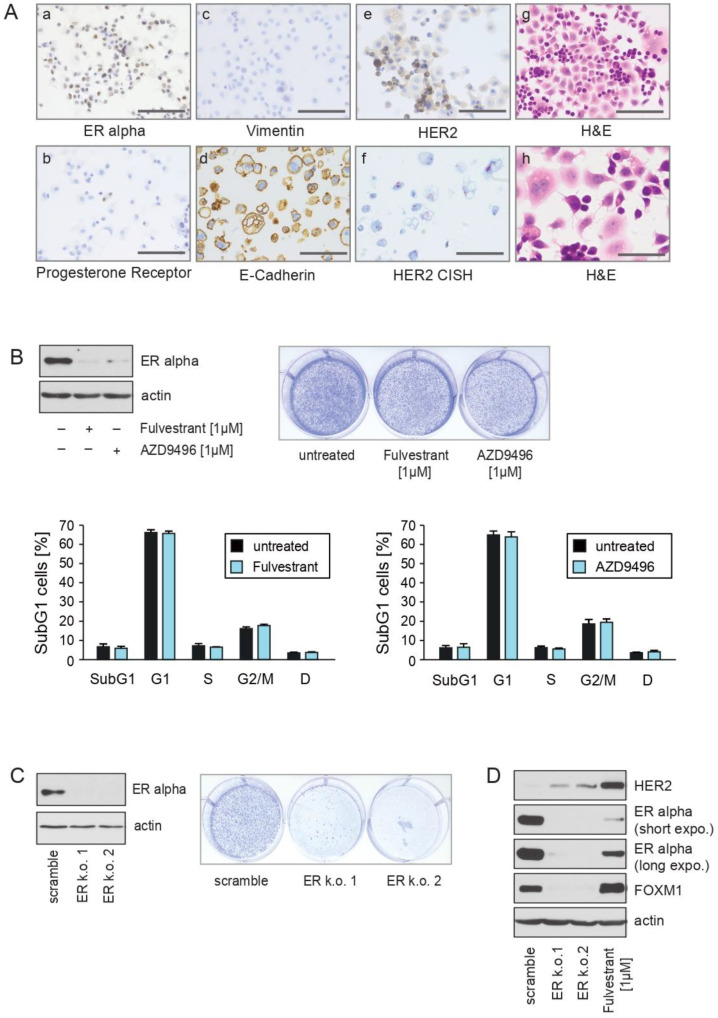
CTC-ITB-01 cells established from a patient with metastatic ER+/HER2− breast cancer show no response to pharmacological inhibition of ER alpha (**A**) (**a**–**e**) Immunohistochemical stainings of formalin-fixed CTC-ITB-01 cells reveal expression of ERα, E-cadherin and modest expression of HER2. Only weak progesterone receptor and no vimentin expression was observed. HER2 CISH shows no amplification of the HER2 gene locus. (**g**,**h**) Hematoxilin and eosin (H&E) staining reveals that cells grow in a mixed epithelial-mesenchymal morphology. (**a**–**e**,**g**) Bar 200 μm (**f**,**h**) Bar 400 μm. (**B**) CTC-ITB-01 cells were cultured in the presence of the indicated fulvestrant or AZD9496 concentrations for 32 h. Western blots of protein lysates were probed with the indicated antibodies (left panel). Equal numbers of CTC-ITB-01 cells were seeded per well of a 6-well plate and treated with the indicated fulvestrant or AZD9496 concentrations. After 9 days cells were fixed and stained (right panel). Equal amounts of CTC-ITB-01 cells were seeded on 6-well culture plates and cultured either with or without the indicated concentrations of fulvestrant or AZD9496. To determine the induction of cell death and/or cell cycle arrest cells were harvested after 36 h. The percentage of SubG1, G1, S, G2/M, D cells was evaluated using propidium iodid staining and flow cytometry. Mean values ± s.d. of four independent experiments are presented (lower panel). (**C**) Stable knockdown of ERα within CTC-ITB-01 cells was performed by using lentiviral transfer of non-targeted or two different targeted shRNAs against ERα. After transduction reduced expression of ERα in CTC-ITB-01 ERα k.o. cells was evidenced by western blotting. Western blots of protein lysates were probed with the indicated antibodies (left panel). Equal numbers of ERα k.o.1, ERα k.o.2 and negative control scramble CTC-ITB-01 cells were seeded on 6-well culture plates. After 9 days cells fixed and stained (right panel). (**D**) To compare the capacity of targeted shRNAs against ERα and fulvestrant to reduce ERα levels equal amounts of lysates from negative control, ERα k.o.1, ERα k.o.2 and fulvestrant treated CTC-ITB-01 cells were analysed by western blotting. Western blots were probed with the indicated antibodies. The uncropped Western Blot Images can be found in Figure S7.

The IHC staining revealed strong ERα, E-cadherin and modest HER2 expression (Figure 1A(a,d,e)). These findings suggest that CTC-ITB-01 cells were derived from ductal carcinoma cells. Only a weak expression of progesterone receptor (PR) in <1% positive cells and no expression of vimentin was observed (Figure 1A(b)). HER2 CISH analysis showed no amplification of the HER2 gene locus (Figure 1A(f)). Hematoxilin and eosin (H&E) staining revealed that the cells grow in a mixed epithelial-mesenchymal morphology as well as adherent and non-adherent fractions (Figure 1A(g,h)). These findings are in accordance with the recently reported features of CTC-ITB-01 [22], indicating a stable phenotype.

To investigate whether CTC-ITB-01 cells are responsive towards pharmacological ERα inhibition, CTC-ITB-01 cells were cultured in the absence or presence of the ER-degraders fulvestrant and AZD9496. The capacity of both drugs to decrease ERα levels within CTC-ITB-01 was monitored by immunoblotting. Both drugs led to decreased levels of ERα (Figure 1B, left panel). Induction of cell death or inhibition of cell growth upon fulvestrant and AZD9496 treatment was assessed using colony-forming assays (Figure 1B, right panel). Notably, no significant differences in the outgrowth of colonies were observed after 9 days with or without treatment. Changes in the distribution of cells within the cell cycle and changes in the amount of SubG1 cells were quantified by propidium-iodide staining and flow cytometry. Consistent with the results from the colony-forming assays, no induction of cell death or cell cycle arrest in CTC-ITB-01 cells could be detected upon fulvestrant or AZD9496 treatment (Figure 1B, lower panel). Collectively, these results indicate that pharmacological inhibition of ERα activity has no significant consequences for the growth or survival of CTC-ITB-01 cells.

To determine the relevance of the ERα for the growth and survival of CTC-ITB-01 cells, we employed stable knockdown of ERα. The efficiency of the shRNAs to inhibit ERα expression within CTC-ITB-01 cells was monitored by immunoblotting (Figure 1C, left panel). The consequences of ERα knockdown upon growth and survival were tested by colony-forming assays (Figure 1C, right panel). Notably, ERα knockdown caused a strong reduction in colony formation, indicating that strategies that lead to a stronger reduction of ERα levels and/or activity might block outgrowth of endocrine resistant breast cancer. We therefore compared the efficacy of fulvestrant with targeted ERα shRNAs to reduce the levels of ERα in CTC-ITB-01 cells. Lysates from non-transduced, shRNA-transduced and fulvestrant-treated cells were analysed by immunoblotting. Interestingly, we observed that stable knockdown caused a much stronger reduction in ERα levels compared to that achieved by fulvestrant treatment (Figure 1D). Notably, treatment of CTC-ITB-01 cells with fulvestrant marginally increased FOXM1 expression, and caused a higher increase in HER2 expression in comparison to ERα knockdown. Several ERα mutations correlate with an attenuated response to endocrine treatment and towards fulvestrant [23]. Interestingly, CTC-ITB-01 cells harbor no ERα mutations [22]. Therefore, the poor response towards ERα inhibition must be mediated through mechanisms other than mutation of ERα.

These findings suggest that therapeutic approaches that lead to stronger reduction of ERα levels and/or ERα activity might have the potential to improve treatment of patients with advanced ER+/HER2− breast cancer. Furthermore, these data also point to a role for HER2 and FOXM1 in resistance to ERα inhibition in ER+ breast cancer cells without HER2 amplification or in the absence of activating HER2 mutations.

### 2.2. The ERα-Degrader Fulvestrant and AZD9496 Inhibit Cell Cycle Progression and FOXM1 Expression in MCF7 but Not in CTC-ITB-01 Cells

**Figure 2:**
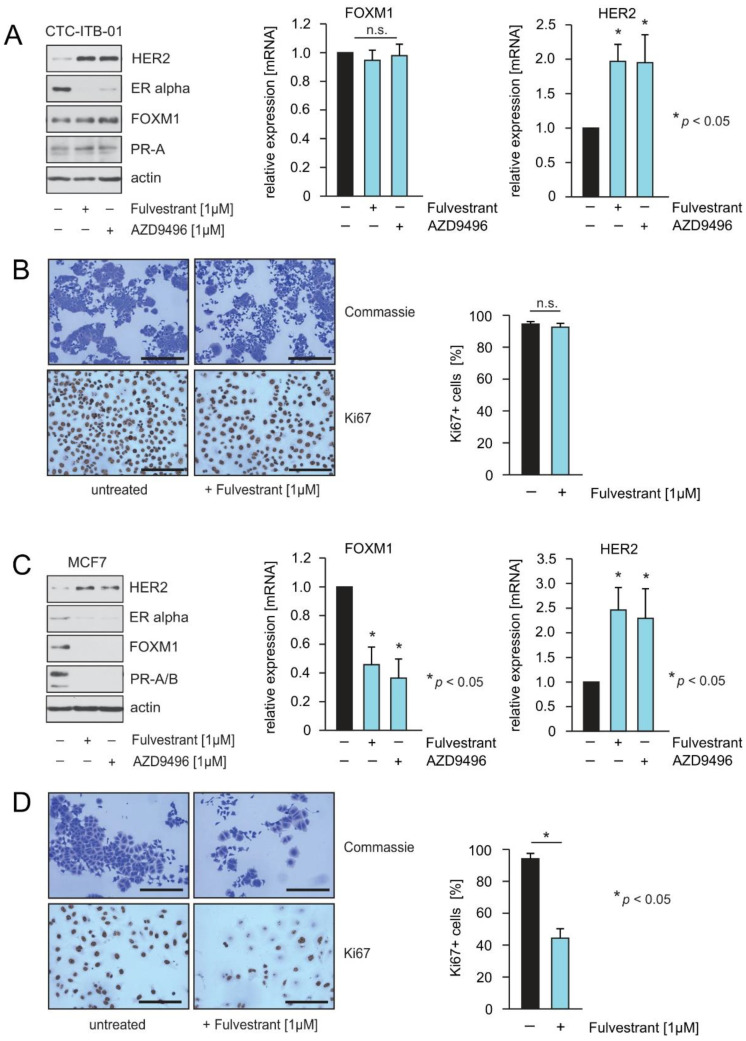
The ERα-degrader fulvestrant and AZD9496 inhibit cell cycle progression and FOXM1 expression within MCF7 but not in CTC-ITB-01 cells. (**A**,**C**) CTC-ITB-01 and MCF7 cells were cultured with or without the indicated concentrations of fulvestrant or AZD9496 for 32 h. Lysates from both cell lines were assayed by immunoblotting for the level of the indicated proteins (left panel). CTC-ITB-01 and MCF7 cells were cultured at equal densities with the indicated concentration of fulvestrant and AZD9496. Cells were harvested after 32 h for mRNA preparation. Expression of FOXM1 and HER2 in non-treated cells relative to treated cells was analysed by qPCR. Mean values ± s.d. of three independent experiments are presented. (Figure 2A,C, middle+right panel). CTC-ITB-01 and MCF7 cells were seeded on cover slides and cultured with or without the indicated concentration of fulvestrant for 32 h. Afterwards cells were fixed and expression of Ki67 was evidenced by immunohistochemical staining (Figure 2B,D, left panel). In parallel slides were stained with commassie to monitor differences in the morphology and density of cells. Quantification of Ki67 positive cells in the non-treated and treated cell (Figure 2B,D, right panel). (**B**,**D**) upper pictures Bar: 400 μm; (**B**,**D**) lower pictures Bar: 200 μm. *p*-values < 0.05 are indicated by asterisks. The uncropped Western Blot Images can be found in Figure S7.

To investigate potential differences in the outcome of fulvestrant and AZD9496 treatment, CTC-ITB-01 and MCF7 cells were cultured in the presence or in the absence of both drugs. Lysates from both cell lines were assayed by immunoblotting to assess the level of the indicated proteins (Figure 2A,C, left panel). Both drugs decreased levels of ERα, and increased levels of HER2 in both cell lines. In MCF7 cells, fulvestrant and AZD9496 led to reduced expression of FOXM1 and progesterone receptor (PR) A+B. In CTC-ITB-01 cells PR expression was unchanged, while FOXM1 expression was marginally increased. CTC-ITB-01 cells displayed only a very slight expression of PR-A, while MCF7 cells expressed higher levels of PR-A and B (Figure 2A,B, left panels). Extremely low expression of PR was also observed in IHC staining, which showed less than 1% PR+ cells (Figure 1A(b)).

FOXM1 and PR are considered to be ERα-target genes that are expressed under the influence of estrogen as long as breast cancer cells are estrogen-responsive [24,25,26]. Sustained expression of FOXM1 in the presence of ERα inhibition in CTC-ITB-01 cells suggests that fulvestrant and AZD9496 cannot suppress ERα activity or that FOXM1 expression is independent of ERα in these cells. To investigate whether fulvestrant and AZD9496 affect FOXM1 and HER2 levels at the transcriptional level, qPCRs were performed (Figure 2A,C, middle + right panel). Potential differences in endocrine treatment responsiveness were investigated. To this end, CTC-ITB-01 and MCF7 cells were seeded on slides and cultured in estrogen-free culture medium either in the absence or presence of fulvestrant. After 28 h cells were fixed and stained for the detection of Ki67 (Figure 2B,D, left panel). The percentage of Ki67 was quantified (right panel). A significant decrease in Ki67+ MCF7 cells was observed, whereas no significant reduction in Ki67 could be observed in CTC-ITB-01 cells upon fulvestrant treatment.

### 2.3. Knockdown Experiments Reveal a Role for HER2 in FOXM1 Expression, Growth and Survival in CTC-ITB-01 Cells

**Figure 3:**
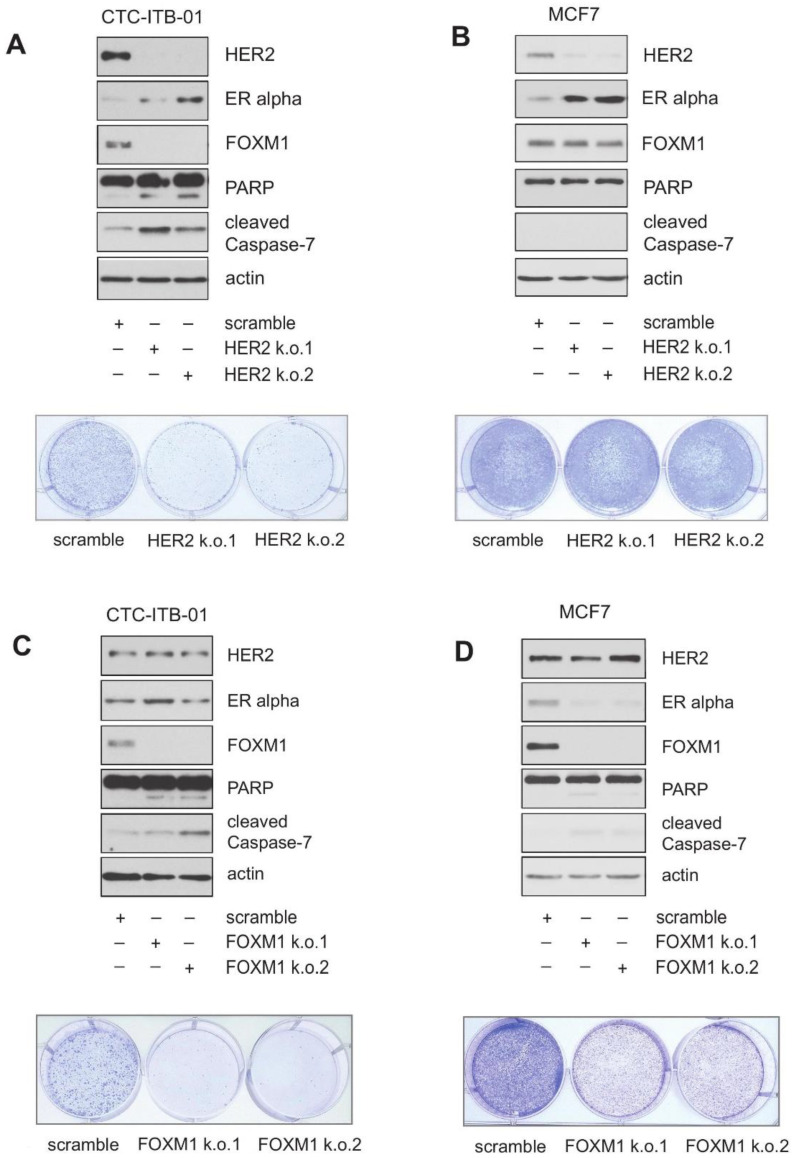
Knockdown experiments reveal a role for HER2 in FOXM1 expression, growth and survival in CTC-ITB-01 cells. (**A**,**B**) Stable knockdown of HER2 within CTC-ITB-01 and MCF7 cells was performed by using lentiviral transfer of non-targeted shRNAs against HER2. After transduction reduced expression of HER2 in CTC-ITB-01 HER2 k.o. and MCF7 HER2 k.o. cells was evidenced by western blotting. Western blots of protein lysates were probed with the indicated antibodies (upper panel). Equal numbers of HER2 k.o.1, HER2 k.o.2 and scramble CTC-ITB-01 and HER2 k.o.1, HER2 k.o.2 and scramble MCF7 cells were seeded on 6-well culture plates. After 9 days cells were fixed and stained (lower panel). (**C**,**D**) Stable knockdown of FOXM1 within CTC-ITB-01 and MCF7 cells was performed by using lentiviral transfer of non-targeted shRNAs against FOXM1. After transduction reduced expression of FOXM1 in CTC-ITB-01 FOXM1 k.o. and MCF7 FOXM1 k.o. cells was evidenced by western blotting (upper panel). Western blots of protein lysates were probed with the indicated antibodies. Equal numbers of FOXM1 k.o.1, FOXM1 k.o.2 and scramble CTC-ITB-01 and FOXM1 k.o.1, FOXM1 k.o.2 and scramble MCF7 cells were seeded on 6-well culture plates. After 9 days cells fixed and stained (lower panel). The uncropped Western Blot Images can be found in Figure S7.

HER2 contributes to endocrine resistance, and patients with HER2 amplified breast cancer have limited endocrine responsiveness [27,28]. To investigate a potential role for HER2 in the resistance of CTC-ITB-01 cells to ERα inhibition and in FOXM1 expression we employed stable knockdown of HER2 in CTC-ITB-01 and MCF7 cells with targeted shRNAs against HER2. In CTC-ITB-01 cells, knockdown of HER2 resulted in a significant reduction in FOXM1 expression, induction of cell death as evidenced by increased cleavage of poly(ADP-ribose)polymerase-1 (PARP1) and caspase-7, as well as a strong reduction in colony formation (Figure 3A). Knockdown of HER2 in MCF7 cells did not reduce FOXM1 expression, and cleavage of PARP1 and caspase-7 was not observed (Figure 3B, upper panel). In addition, no significant decrease in colony formation upon HER2 knockdown was detected in MCF7 cells (Figure 3B, lower panel). These results demonstrate that in CTC-ITB-01 cells FOXM1 expression, cell growth and survival depend on HER2, whereas in MCF7 cells FOXM1 expression depends on ERα and not on HER2. Interestingly, both cell lines display increased expression of ERα upon HER2 knockdown. These observations point to the existence of a crosstalk between HER2 and ERα in both cell lines.

To evaluate the importance of FOXM1 for growth and survival, stable knockdown of FOXM1 was employed in CTC-ITB-01 and MCF7 cells. Both cell lines depend on FOXM1 for growth and survival. In CTC-ITB-01 cells, FOXM1 knockdown induced cell death as monitored by PARP1 and caspase-7 cleavage and through colony-forming assays (Figure 3C). In MCF7 cells, FOXM1 knockdown led to a slight increase in PARP1 and caspase-7 cleavage (Figure 3D, upper panel) and decreased outgrowth of colonies (Figure 3D, lower panel). Reduced expression of FOXM1 led to decreased expression of ERα in MCF7 cells, as also described by others [29], consistent with a complex regulatory loop between ERα and FOXM1 [24,29]. Interestingly, knockdown of FOXM1 did not reduce expression of ERα in CTC-ITB-01 cells (Figure 3C, upper panel). This result shows that in CTC-ITB-01 cells, ERα expression is sustained in the absence of FOXM1, and underlines differences between CTC-ITB-01 and MCF7 cells. We also observed that knockdown of FOXM1 in MCF7 cells caused less reduction in colony formation compared to CTC-ITB-01 (Figure 3C, lower panel), as well as induction of senescence as monitored by typical morphological changes. In CTC-ITB-01 cells we observed fewer cells with senescence-like morphology, indicating that FOXM1 knockdown mainly causes induction of senescence in MCF7 cells, while predominantly causing apoptosis in CTC-ITB-01 cells (Figure S2).

### 2.4. Pharmacological HER2 Inhibition Has Different Effects in CTC-ITB-01 and MCF7 Cells

**Figure 4:**
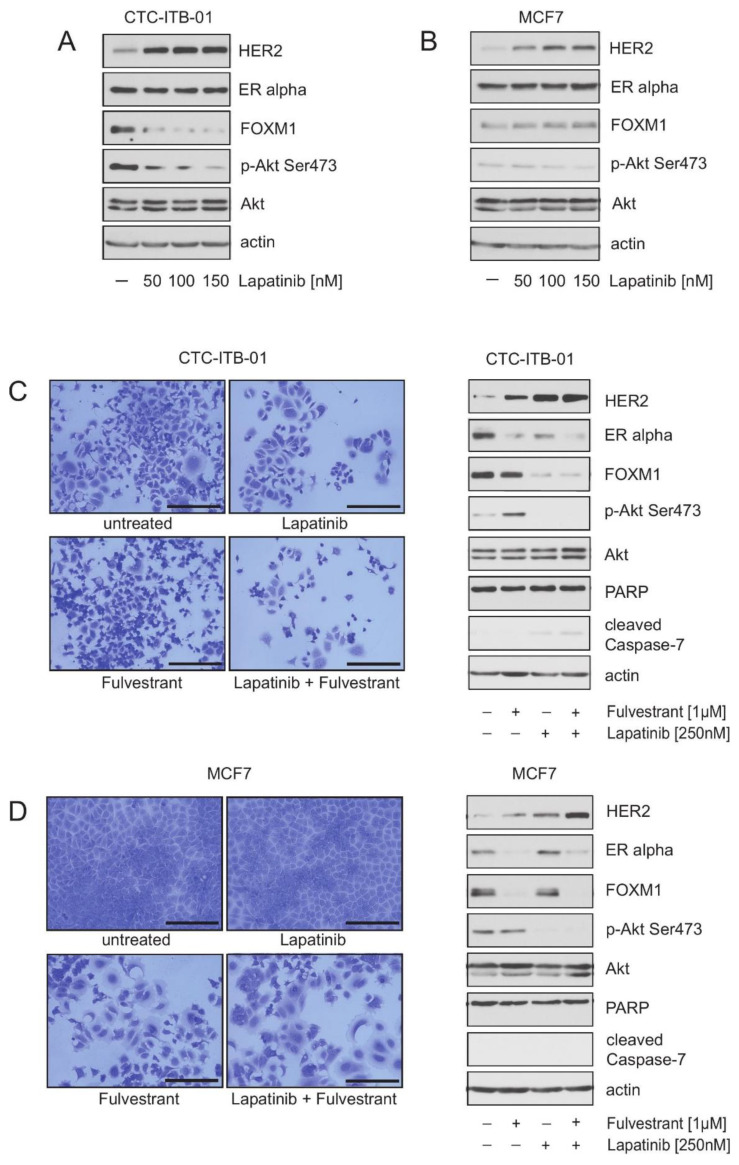
Pharmacological HER2 inhibition has different effects in CTC-ITB-01 and MCF7 cells. (**A**,**B**) CTC-ITB-01 and MCF7 cells were cultured with or without the indicated concentrations of lapatinib for 32 h. Lysates from both cell lines were assayed by immunoblotting for the level of the indicated proteins. (**C**,**D**) CTC-ITB-01 and MCF7 cells were cultured at equal densities with the indicated concentration of lapatinib and fulvestrant alone or in combination. Cells were harvested after 32 h for western blotting. Western blots of protein lysates were probed with the indicated antibodies (right panel). (**C**,**D**) Equal numbers of CTC-ITB-01 and MCF7 cells were seeded on 6-well culture plates and cultured with or without the indicated concentration of lapatinib and fulvestrant. After 9 days cells fixed and stained with commassie to monitor differences in the morphology and density of cells. Macroscopic photos are shown of the fixed cell colonies for CTC-ITB-01 and MCF7 cells treated with lapatinib (250 nM), fulvestrant (1 μM) and lapatinib (250 nM) plus fulvestrant (1 μM) (left panel). Bar 200 μm. The uncropped Western Blot Images can be found in Figure S7.

Motivated by our observation that HER2 plays a role in FOXM1 expression and survival in CTC-ITB-01 cells, we tested the susceptibility of CTC-ITB-01 cells to pharmacological inhibition of HER2. Treatment of CTC-ITB-01 and MCF7 cells with increasing concentrations of lapatinib resulted in a dose-dependent decrease in FOXM1 expression in CTC-ITB-01 cells (Figure 4A), whereas in MCF7 cells FOXM1 expression was not reduced in the presence of lapatinib (Figure 4B). Lapatinib treatment also caused a dose-dependent reduction in p-Akt^Ser473^ and increased expression of HER2 in both cell lines (Figure 4A,B). These findings are in accordance with our previous results from the HER2 knockdown experiments, which showed the HER2 dependence of FOXM1 expression in CTC-ITB-01 cells but not in MCF7 cells (Figure 3A,B, upper panels).

The observations that CTC-ITB-01 and MCF7 cells display increased expression of HER2 upon ERα inhibition (Figure 1D and Figure 2A,C) and that knockdown of HER2 leads to increased expression of ERα (Figure 3A,B, upper panels) point to the existence of a crosstalk between ERα and HER2 in both cell lines. Experimental studies as well as results from clinical trials show that HER2 and ERα synergize to escape from both anti-ERα and anti-HER2-targeted therapies [30,31,32]. Therefore, we investigated next whether crosstalk between ERα and HER2 contributes to the attenuated response of CTC-ITB-01 cells towards ERα inhibition and/or HER2 inhibition.

To determine whether lapatinib alone or in combination with fulvestrant has the potential to inhibit outgrowth or to induce cell death, we cultured CTC-ITB-01 and MCF7 cells either in the presence of lapatinib, fulvestrant or both drugs in combination as indicated. After treatment, all cells were either harvested and lysed for western blotting, or fixed and stained with Coomassie at equivalent time points. Immunoblotting revealed that lapatinib either alone or in combination with fulvestrant caused decreased expression of FOXM1 and a slight increase in cleaved caspase-7 in CTC-ITB-01 cells (Figure 4C, right panel). Consistently, in colony-forming assays lapatinib and lapatinib plus fulvestrant led to only a slightly reduced number of cells in comparison to non-treated and fulvestrant treated CTC-ITB-01 cells (Figure 4C, left panel). Although lapatinib-treated CTC-ITB-01 cells displayed a modest increase in cleaved caspase-7 and a reduced number of cells in colony-forming assays in comparison to their untreated counterparts, the effects of lapatinib on CTC-ITB-01 cells were only modest (Figure 4C) and the reduction of FOXM1 was not as strong as in the HER2 knockdown experiments (Figure 3A). Notably, these findings are possibly in line with the results from the CALGB 40302 (Alliance) trial, which reported that addition of lapatinib to fulvestrant treatment did not improve progression free survival (PFS) or overall survival (OS) in advanced ER+ breast cancer [33].

Interestingly, CTC-ITB-01 cells displayed a strong increase in HER2 levels in the presence of lapatinib (Figure 4C, right panel), indicating that rescue mechanisms may exist that compensate for pharmacological HER2 inhibition. Notably, CTC-ITB-01 cells have no HER2 mutations [22] and therefore aberrant function of HER2 is not an explanation for the modest response towards lapatinib. Immunoblotting of lysates from MCF7 cell show decreased expression of FOXM1 only in the presence of fulvestrant, whereas lapatinib did not reduce expression of FOXM1 (Figure 4B,C, right panel). Consistently, in colony-forming assays reduced numbers of cells could only be observed in the presence of fulvestrant (Figure 4C, left panel). Although lapatinib treatment only slightly reduced FOXM1, these findings still confirm that FOXM1 expression is HER2-dependent and ERα-independent in CTC-ITB-01 cells, whereas in MCF7 cells expression of FOXM1 is ERα-dependent and HER2-independent.

### 2.5. Treatment of CTC-ITB-01 Cells with the Proteasome Inhibitor Carfilzomib Causes Inhibition of ER alpha, FOXM1 and HER2 Expression and Induction of Cell Death

**Figure 5:**
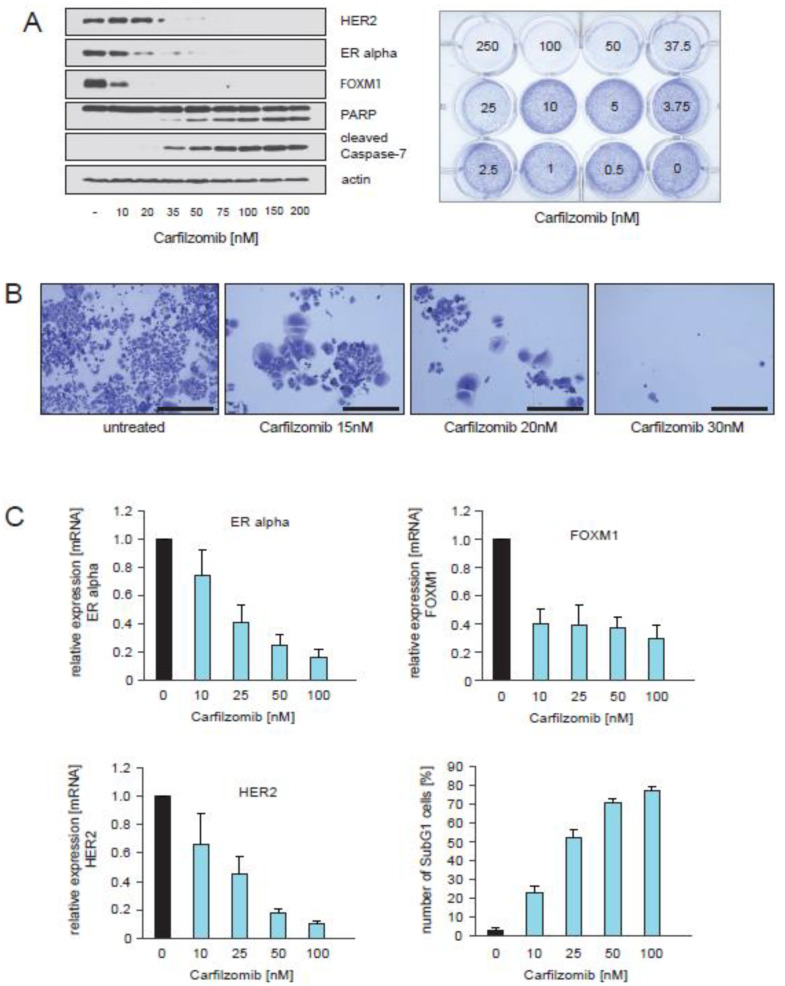
Treatment of CTC-ITB-01 cells with the proteasome inhibitor carfilzomib causes inhibition of ER alpha, FOXM1 and HER2 expression and induction of cell death (**A**) CTC-ITB-01 cells were cultured with or without the indicated concentrations of carfilzomib for 32 h. Western blots of protein lysates were probed with the indicated antibodies (left panel). Equal numbers of CTC-ITB-01 cells were seeded on 12-well culture plates and treated with the indicated carfilzomib concentrations. After 9 days cells were fixed and stained (right panel). (**B**) Macroscopic photos are shown of the fixed cell colonies for CTC-ITB-01 cells either untreated or treated with the indicated carfilzomib concentration. Bar 400 μm. (**C**) CTC-ITB-01 cells were cultured at equal densities with the indicated concentration of carfilzomib. Cells were harvested after 32 h for mRNA preparation. Expression of FOXM1 and HER2 in non-treated cells relative to treated cells was analysed by qPCR. Mean values ± s.d. of three independent experiments are presented (upper panel + lower left panel). To determine the induction of cell death relative to the applied carfilzomib concentrations equal numbers of CTC-ITB-01 cells were seeded on 12-well culture plates and cultured in the absence or in the presence of the indicated concentrations for 36 h. The percentage of SubG1 cells was evaluated using propidium iodide staining and flow cytometry. Mean values ± s.d. of three independent experiments are presented (lower right panel). The uncropped Western Blot Images can be found in Figure S7.

The above findings raise the question as to whether direct targeting of HER2 with established HER2 inhibitors in ER+/HER2− breast cancer may be therapeutically useful, and whether other strategies might be more promising instead. We and others have reported that proteasome inhibitors (PIs) inhibit the expression of FOXM1 in breast cancer and other types of cancer [34,35,36,37]. Furthermore, we have observed that similar to PI treatment, knockdown of FOXM1 in MCF7 cells caused down-regulation of numerous genes whose expression correlates with resistance to endocrine therapy, decreased sensitivity to chemotherapy, poor prognosis and decreased metastasis-free survival in ERα+ breast cancer patients [34]. In addition, we found that PIs inhibit ERα expression and disrupt signaling pathways that mediate resistance to commonly used treatment regimens in ER+/HER2− breast cancer [34], and that PIs inhibit HER2 through multiple mechanisms [34,35]. The results of these previous findings prompted us to investigate the consequence of the second generation PI carfilzomib on HER2, ERα and FOXM1 expression in CTC-ITB-01 cells using western blotting and qPCR (Figure 5A,C). Induction of apoptosis upon carfilzomib treatment was assessed by PARP1 and caspase-7 cleavage, the use of colony-forming assays (Figure 5A), and by quantification of the SubG1 DNA content in treated cells (Figure 5C, lower right panel). Immunoblotting demonstrated that carfilzomib caused decreased expression of ERα, FOXM1 and HER2 in CTC-ITB-01 cells. These findings are in accordance with results that we reported previously for MCF7 cells [35]. Notably, in contrast to ERα and HER2, down-regulation of FOXM1 expression under carfilzomib treatment was not dose-dependent, suggesting that FOXM1 expression may already be blocked at low carfilzomib concentrations (Figure 5A, left panel, Figure 5C, upper right panel).

In the circulation, CTCs can exist as single cells that are essentially in suspension. CTCs can also form clusters through adhering to each other. CTC clusters are associated with a poor clinical outcome, probably due to the survival advantage provided to CTCs in clusters [38]. Adhesion to substrate is also required for CTCs to form metastases at secondary sites. We therefore determined whether the cultivation of breast cancer cells under adherent or suspension conditions modifies their properties. We found that CTC-ITB-01 cells can survive in suspension, in contrast to MCF7 cells. ERα expression was reduced in both CTC-ITB-01 and MCF7 cells grown in suspension compared to cells grown under adhesive conditions, indicating that ERα expression is regulated by adhesion in both cell lines. However, CTC-ITB-01 cells sustained FOXM1 expression when cultured in suspension in contrast to MCF7 cells (Figure S3), consistent with our observation that CTC-ITB-01 cells sustain FOXM1 expression independently of ERα. Interestingly, CTC-ITB-01 cells exhibited increased levels of activated p-Akt ^S473^ when cultured in suspension, indicating a possible mechanism that helps the cells to survive in the blood or lymphatic systems (Figure S3). Nevertheless, treatment of CTC-ITB-01 cells with carfilzomib led to cell death regardless of whether the cells were cultured adherently or in suspension (Figure 5, Figure S4), underscoring the potential of carfilzomib to target endocrine resistant CTCs.

### 2.6. HER2 and FOXM1 Expression within CTC-ITB-01 Cells Depends on NFkB-Signaling

**Figure 6:**
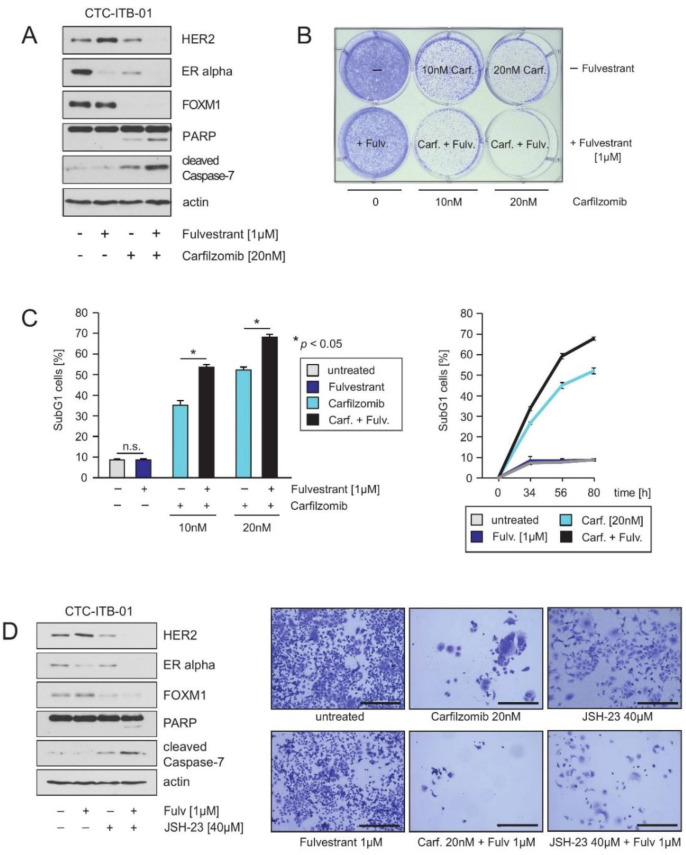
HER2 and FOXM1 expression within CTC-ITB-01 cells depends on NFkB-signaling. (**A**) CTC-ITB-01 cells were cultured in the absence or in the presence of the indicated concentrations and combinations of carfilzomib and fulvestrant. After 32 h cells were harvested. Western blots of the protein lysates were probed with the indicated antibodies. (**B**) To determine the induction of cell death in response to the indicated drug combinations equal numbers of CTC-ITB-01 cells were seeded on 6-well culture plates and cultured in the absence or in the presence of the indicated drug concentrations. After 9 days cells were fixed and stained. (**C**) To determine the induction of cell death in response to the indicated drug combinations equal numbers of CTC-ITB-01 cells were seeded on 6-well culture plates and cultured in the absence or in the presence of the indicated drug concentrations. The percentage of SubG1 cells was evaluated after 80 h using propidium iodide staining and flow cytometry. Mean values ± s.d. of three independent experiments are presented (left panel). The percentage of SubG1 cells was evaluated at different time points using propidium iodide staining and flow cytometry. Mean values ± s.d. of three independent experiments are presented (right panel). (**D**) CTC-ITB-01 cells were cultured in the absence or in the presence of the indicated concentrations and combinations of carfilzomib and fulvestrant. After 32 h cells were harvested. Western blots of the protein lysates were probed with the indicated antibodies (left panel). Equal numbers of CTC-ITB-01 cells were seeded on 6-well culture plates and cultured either with or without the indicated drug combinations. After 9 days cells were fixed and stained (right panel). Macroscopic photos are shown of the fixed cell colonies for CTC-ITB-01 cells either untreated or treated with fulvestrant (1 μM), carfilzomib (20 nM), carfilzomib (20 nM) plus fulvestrant (1 μM), JSH-23 (40 μM) and JSH-23 plus fulvestrant (1 μM). Bar 400 μm. The uncropped Western Blot Images can be found in Figure S7.

Several reports describe a role for NFkB for endocrine therapy resistance, including to fulvestrant [39]. Consistently, inhibition of NFkB can restore sensitivity to fulvestrant [40]. PIs were originally developed as inhibitors of the NFkB signaling pathway and thus, the clinical efficiency of PIs was first attributed to their ability to suppress NFkB [41,42]. With this in mind, we investigated whether treatment with carfilzomib sensitizes CTC-ITB-01 cells towards ERα inhibition and fulvestrant. Cultivation of CTC-ITB-01 cells with carfilzomib, either alone or in combination with fulvestrant, inhibited outgrowth and induced cell death as monitored by PARP1 and caspase-7 cleavage, colony-forming assays and quantification of SubG1 cells (Figure 6A–C). Notably, in the presence of carfilzomib plus fulvestrant, inhibition of colony outgrowth and induction of cell death was significantly higher than with carfilzomib treatment alone. These data suggest that PIs have the ability to sensitize CTC-ITB-01 cells to fulvestrant. Importantly, the combination of carfilzomib plus fulvestrant augmented suppression of HER2 and ERα compared to carfilzomib alone, and increased induction of cell death as monitored by increased PARP1 and caspase-7 cleavage (Figure 6A). We conclude that regimens that more strongly suppress ERα might be useful in targeting endocrine resistant ER+ breast cancer cells, and that drug combinations that target the ERα through multiple mechanisms might be a promising strategy to target ERα in endocrine resistant breast cancer cells.

PIs inhibit numerous other proteins in addition to NFkB, and might suppress FOXM1 expression through stabilization of proteins that repress FOXM1 mRNA and protein expression [43,44]. To investigate whether carfilzomib suppresses HER2, ERα and FOXM1 expression through inhibition of NFkB signaling or through other mechanisms, CTC-ITB-01 cells were cultured in the presence or absence of the NFkB inhibitor JSH-23 [44]. Control experiments verified that treatment with JSH-23 resulted in exclusion of p65 from the nucleus (Figure S5A–C). Treatment of CTC-ITB-01 cells with JSH-23 either alone or in combination with fulvestrant suppressed HER2, ERα and FOXM1 protein expression, and induced cell death similar to carfilzomib (Figure 6D, left panel + right panel). JSH-23 also reduced transcription of FOXM1 (Figure S6). These data suggest that inhibition of NFkB is the main mechanism through which carfilzomib suppresses HER2, ERα and FOXM1 expression. 

**Figure 7:**
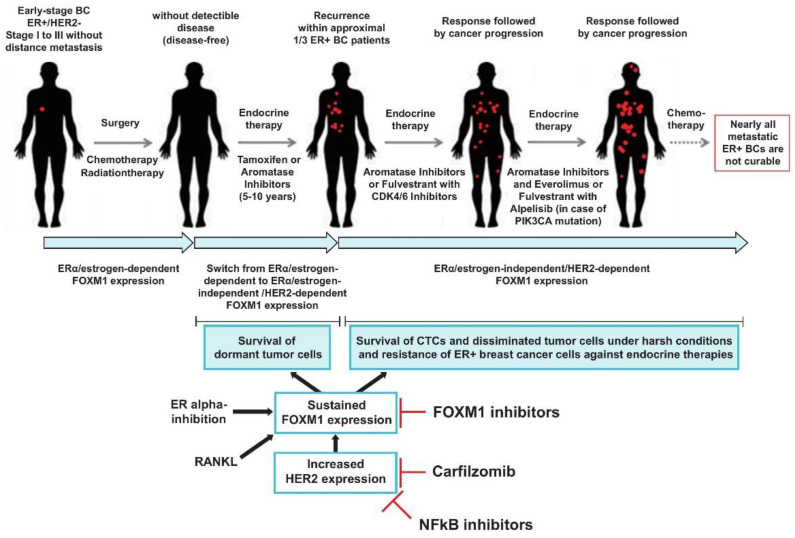
Proposed model how switching from ERα-dependent to HER2-dependent/ERα-independent expression of FOXM1 enables disseminated ER+/HER2− breast cancer cells to re-initiate tumor cell growth and metastasis formation in the presence of endocrine treatment. Progression of ER+/HER2− breast cancer proceeds through multiple stages. Although ER+/HER2− breast cancer is associated with good prognosis around one third progress and develop late recurrence sometimes even after decades. Endocrine therapy in patients with ERα+ breast cancer has significantly improved patient outcomes. However resistance towards endocrine therapies occurs often and is found in nearly all hormone receptor positive breast cancer patients with metastatic disease. Despite insights into mechanisms of endocrine therapy resistance and the development of new strategies for treatment of endocrine resistant ER+/HER2− metastatic breast cancers such as mTOR (everolimus), PIK3CA (alpelisib) and CDK4/6 inhibitors, the management of advanced hormone-receptor positive breast cancer with resistance to endocrine therapies as well as strategies to avoid late recurrence remains a significant challenge. Mechanisms of acquired endocrine resistance and late recurrence in patients with ER+/HER2− breast cancer are complex and not fully understood. The results of our experiments show that CTCs are resistant towards ERα inhibition and reveal a particular relevance of HER2 for sustained FOXM1 expression even in ER+ breast cancer without HER2 amplification or without activating HER2 mutations. Our results point to a switch from ERα-dependent to HER2-dependent and ERα-independent expression of FOXM1 when patients progress under endocrine treatment. Given that FOXM1 is known to be involved in endocrine resistance, expansion of stem-like cancer cells, stem cell self-renew, cancer initiation and metastasis, it is conceivable that switching from ERα-dependent to ERα-independent expression of FOXM1 enables disseminated ER+/HER2− to re-initiate tumor cell growth and metastasis formation in the presence of endocrine treatment such as observed in patients with late recurrence and metastatic ER+/HER2− breast cancer. Our results also demonstrate that inhibition of the NFkB signaling pathway suppresses HER2 and FOXM1 expression and causes cell death within CTCs. These findings suggest that targeting of NFkB and FOXM1 might be possible therapeutic approaches to prevent late recurrence and to treat endocrine resistance.

Collectively, these results suggest that HER2 and FOXM1 expression in CTC-ITB-01 cells depends on NFkB. Targeting of NFkB and FOXM1 might therefore allow endocrine treatment resistance to be circumvented and might be beneficial for the treatment of metastatic ER+/HER2− breast cancer (Figure 7).

## 3. Discussion

Around 70% of breast cancers are positive for ERα, and the targeting of ERα with tamoxifen or aromatase inhibitors (AIs) is the standard treatment for patients with ER+ breast cancer [2]. The translational strategy of long-term anti-hormone adjuvant therapy to target ERα activity has efficiently improved patient outcome [4]. However, acquired resistance to anti-hormone therapy is an inevitable challenge after prolonged therapies [1]. Understanding the mechanisms that underlie this resistance will allow strategies to be developed for overcoming it [8].

The data we present here suggest that CTCs from patients with acquired endocrine resistance are useful for functional studies that investigate the mechanisms that underlie anti-hormone therapy resistance. CTC-ITB-01 and MCF7 cells exhibit significant differences in their ability to respond to ERα inhibition under estrogen-free conditions (Figure 2A–D). CTC-ITB-01 cells are refractory to treatment with the ERα-degraders fulvestrant and AZD9496 (Figure 1C and Figure 2A,B), suggesting that CTC-ITB-01 cells are truly endocrine therapy resistant and therefore suitable for functional studies to investigate potential mechanisms connected with and attenuated response towards ERα inhibition. Nevertheless, although CTC-ITB-01 cells do not respond to the ERα-degraders fulvestrant and AZD9496 (Figure 1B and Figure 2B), knockdown of the ERα led to a strong reduction of CTC-ITB-01 cells in colony-forming assays, indicating that ERα is still relevant for growth of CTC-ITB-01 cells (Figure 1C, right panel). As CTC-ITB-01 cells do not harbor activating mutations in the ERα [22], this raises the question as to whether the therapeutic efficacy of SERDs such as fulvestrant or AZD9496 is limited due to insufficient inhibition of ERα levels and/or ERα activity, and whether treatment of ER+/HER2− breast cancer could be improved by the use of stronger ERα inhibitors.

Our results suggest that CTC-ITB-01 cells are still dependent on ERα (Figure 1C). This conclusion is in line with clinical data showing that ER+ breast cancers frequently remain dependent on ERα signaling even after acquiring resistance to endocrine treatment. This persistent ERα dependence has stimulated efforts to further optimize ERα-targeting drugs [45]. However, recently published data show that prospective optimization of ERα degradation, particularly with a strong reliance on MCF7 cells, is not sufficient for the identification of ligands that consistently fully antagonize ERα transcriptional activity [45]. Thus, the use of other cell culture models such as cell lines derived from CTCs isolated from patients with acquired endocrine resistance, might be more suitable for the development of new, more powerful ERα-targeting compounds.

A strong positive correlation between HER2 overexpression and FOXM1 has been reported in breast cancer cell lines and in breast cancer samples with HER2 amplification [46]. Treatment of HER2-positive breast cancer with the HER2/EGFR inhibitor lapatinib leads to decreased FOXM1 expression, suggesting that HER2 positively regulates FOXM1 [37,46]. Our findings also demonstrate that HER2 in CTC-ITB-01 cells is relevant for cell growth, FOXM1 expression and survival (Figure 3A and Figure 4A,C), despite that fact that CTC-ITB-01 cells display no HER2 amplification (Figure 1A(f)) or activating HER2 mutations [20]. However, although CTC-ITB-01 cells show a strong dependence on HER2 in knockdown experiments (Figure 3A), pharmacological inhibition of HER2 with lapatinib either alone or in combination with fulvestrant only modestly reduced colony formation and FOXM1 expression in comparison to HER2 knockdown (Figure 3A and Figure 4A,C). These findings raise the question as to whether pharmacological targeting of HER2 with lapatinib is powerful enough to block HER2 activity sufficiently, or whether adaptive rescue mechanisms exist that enable CTC-ITB-01 cells to circumvent HER2 inhibition. 

Increased levels of HER2 were observed upon fulvestrant treatment, indicating a potential crosstalk between HER2 and ERα in CTC-ITB-01 and MCF7 cells (Figure 1D and Figure 2A,C). Moderate up-regulation of HER2 in ER+/HER2− breast cancer cell lines was also observed by others in MCF7 cells subjected to long-term estrogen deprivation and tamoxifen treatment suggesting that this increased expression of HER2 might be responsible for attenuated responses to estrogen withdrawal and tamoxifen [47]. Cell culture-based experiments with these modified MCF7 cells have shown that inhibition of HER2 with lapatinib restored endocrine sensitivity [47]. However, the results from the CALGB 40302 (Alliance) trial demonstrated that addition of lapatinib to fulvestrant did not improve progression free survival (PFS) or overall survival (OS) in advanced ER+/HER2− breast cancer [33]. This observation suggests that tumor cells from metastatic ER+/HER2− breast cancer are less reliant on HER2 than would be expected from the results of cell culture experiments with modified MCF7 cells, or that metastatic ER+/HER2− breast cancer cells have the ability to circumvent or to compensate for lapatinib-mediated HER2 inhibition. Collectively, our results suggest that CTC-ITB-01 cells might better reflect the behavior of endocrine resistant ER+/HER2− breast cancer cells in metastatic breast cancer patients than modified MCF7 cells.

CTC-ITB-01 cells display strongly increased HER2 levels in the presence of lapatinib (Figure 4C, right panel), indicating that potential rescue mechanisms may exist that compensate for pharmacological HER2 inhibition in ER+/HER2− breast cancer cells. CTC-ITB-01 cells have no HER2 mutations and therefore aberrant function of HER2 is not an explanation for the modest response to lapatinib. One often observed event in ER+/HER2− endocrine resistant breast cancers are mutations within the PI3K/Akt/mTOR axis, leading to a persistent activation of this HER2 down-stream pathway [7]. Activating mutations in this pathway should result in activation of this signaling pathway even in the presence of HER2 inhibition. However, we observed decreased levels of p-Akt^Ser473^ in CTC-ITB-01 and MCF7 cells in the presence of lapatinib (Figure 4A–D) suggesting that both cell lines might have the possibility of switching to another alternative pathway, or that lapatinib is not powerful enough to suppress the PI3K/Akt/mTOR axis completely in these cells.

Our results indicate that HER2 and FOXM1 expression in endocrine resistant CTC-ITB-01 cells is dependent on NFkB signaling, consistent with the observation that acquired endocrine resistance is associated with the constitutive activation of inflammation-associated transcription factors such as NFkB [48,49]. Several reports show a role for NFkB in the development of endocrine resistance through various mechanisms [50]. Up-regulation of NFkB activation is able to drive fulvestrant resistance [39], and NFkB inhibition restores sensitivity towards fulvestrant [40], which can be explained in part by a trans-repressive interaction between ERα and NFkB [50]. Thus, ERα can prevent binding of NFkB to the DNA. NFkB activity is countered by the increased interaction with co-repressors and a competition for co-activators with ERα. Inhibition of ERα causes subsequent activation of NFkB and increased expression of NFkB target genes [50]. Furthermore, NFkB can directly stimulate FOXM1 transcription [51,52,53]. As FOXM1 can activate transcription of ERα [29], NFkB-stimulated FOXM1 expression represents an indirect mechanism through which NFkB can additionally regulate ERα transcription.

HER2 and NFkB mutually reinforce each other’s expression. HER2 activates NFkB through activation of Akt/IKK/IkBalpha. Subsequently, NFkB binds to the HER2 promoter and activates HER2 expression, establishing a positive feedback loop that sustains activation of NFkB and HER2 expression [54]. As ERα has the ability to repress HER2 expression through binding to the HER2 promoter [55,56,57], inhibition or loss of ERα through fulvestrant treatment leads to increased HER2 expression and as a consequence activation of NFkB. In addition, HER2 expression is transiently activated in luminal breast cancer stem cells (CSCs), through the RANKL/RANK-induced activation of NFkB. This NFkB-mediated transient HER2 expression was shown to be relevant for endocrine resistance and survival of CSCs in the bone marrow in animal models [54].

Here we show that carfilzomib and the NFkB inhibitor JSH-23 suppress HER2, ERα and FOXM1 expression (Figure 6A,D). In colony-forming assays, carfilzomib reduced colony formation more markedly than JSH-23. Although both drugs cause a strong reduction in HER2 levels, carfilzomib can additionally suppress HER2 activity in an NFkB-independent manner through stabilization of the HER2-specific phosphatase BDP1 [35]. Thus, although the suppression of HER2, ERα and FOXM1 expression is connected to NFkB inhibition, PIs have a stronger impact on HER2 than would be expected through NFkB inhibition alone, consistent with suppression of HER2 expression plus inhibition of HER2 activity by PIs [34,35].

Collectively, our results show profound differences between CTC-ITB-01 and MCF7 cells with regard to FOXM1 expression in the presence of ERα inhibitors (Figure 2A,C) or under estrogen deprivation [22]. MCF7 cells display a strong reliability on estrogen and ERα, whereas CTC-ITB-01 cells are dependent on HER2 for FOXM1 expression (Figure 3A,B). These findings point to a switch from ERα-dependent to HER2-dependent/ERα-independent expression of FOXM1 when patients progress under endocrine treatment, even in ER+ breast cancer without HER2 amplification or without activating HER2 mutations (Figure 7). Given that FOXM1 is involved in endocrine resistance, expansion of stem-like cancer cells, stem cell self-renewal, cancer initiation and metastasis [5,6] it is conceivable that a switch from ERα-dependent to ERα-independent/HER2-dependent expression of FOXM1 enables disseminated ER+/HER2− to re-initiate tumor cell growth and metastasis formation in the presence of endocrine treatment, as is observed in patients with late recurrence and metastatic ER+/HER2− breast cancer. These findings suggest that targeting of NFkB and FOXM1 might be an efficient therapeutic approach to prevent late recurrence and to treat endocrine resistance (Figure 7). Consistently, panepoxydone inhibits NFkB and FOXM1 and exhibits a strong anti-tumor activity against breast cancer cell lines including ER+/HER2− MCF7 cells [52], underlining the relevance of NFkB and FOXM1 as targets in ER+/HER2− breast cancer. In this regard it is also notable that newly-developed compounds for specifically targeting of FOXM1 have been recently reported [58]. Such compounds might be useful either alone or in combination with other drugs to target ER+/HER2− breast cancer cells.

We show in this study that CTC-ITB-01 cells can efficiently be targeted through inhibition of the NFkB signaling pathway by the PI carfilzomib. Carfilzomib suppresses HER2, ERα and FOXM1 expression (Figure 5A,C). In combination with fulvestrant, the ability of carfilzomib to suppress HER2 and ERα expression is increased (Figure 6A). As lapatinib only weakly suppressed colony formation and poorly induced apoptosis in CTC-ITB-01 cells, our results suggest that direct targeting of HER2 might be not powerful enough to have a therapeutic impact on metastatic ER+/HER2− breast cancer cells (Figure 4C). Furthermore, these cells may also harbor adaptive mechanisms or further mutations that enable them to circumvent pharmacological inhibition of HER2. The results of this study show that targeting NFkB with PIs such as carfilzomib in combination with fulvestrant in CTCs from ER+/HER2− breast cancer might offer the opportunity to block metastatic progression at a subclinical stage before metastases appear.

An important limitation of this study that needs to be considered when assessing the broader applicability of the results presented in this paper is that the study was performed with CTCs from only a single patient. Clearly it is important that further functional studies are performed to investigate acquired endocrine resistant mechanisms using CTCs from additional patients. This will be the focus of future work. Nevertheless, CTCs exhibiting expression of HER2 are frequently observed in metastatic breast cancer patients who originally had ER+/HER2− primary tumors [59]. This suggests that the CTC cell line CTC-ITB-01 may perhaps be representative of at least a proportion of CTCs from patients with ER+/HER2− metastatic breast cancer. Consistently, the multi-center DETECT-III trial was designed to evaluate the efficacy of lapatinib in targeting HER2+ CTCs in metastatic ER+/HER2− breast cancer patients [60]. In this regard, it is significant that an experimental study with CTCs from patients with ER+/HER2− metastatic breast cancer showed that acquisition of HER2 does not generally indicate HER2 oncogene dependence and drug susceptibility. Instead, cultured CTCs maintain HER2+ and HER2− subpopulations that interconvert spontaneously. Although both subpopulations show comparable tumor-initiating potential, they can be distinguished through differences in proliferation rates, oxidative stress and cytotoxic chemotherapy resistance. This suggests that the interconverting phenotypes within patient-derived CTCs may contribute to progression of breast cancer and acquisition of drug resistance [59]. The existence of HER2+ and HER2− subpopulations that can interconvert suggests underlying tumor cell plasticity in these advanced patient-derived breast CTCs, rather than a hierarchical cancer stem-cell model as described in drug-resistant subpopulations within established breast cancer cell lines [59] and may therefore explain the differences between CTC-ITB-01 and MCF7 cells within our experiments. Although we also observed that CTC-ITB-01 cells show less susceptibility to lapatinib, we observed that knockdown of HER2 led to a strong reduction in colony forming ability, induction of apoptotic cell death, and decreased expression of FOXM1 (Figure 3A). These findings indicate a strong dependence on HER2 for proliferation and survival. The DETECT-III trial will show to what extent HER2+ CTCs in ER+/HER2− patients are susceptible towards HER2 inhibition with lapatinib.

In summary, we demonstrate here that CTCs are useful for testing the efficiency of potential new drugs aimed at circumventing therapy resistant mechanisms. Using such cells, we have obtained many insights into endocrine resistant mechanisms that are not found in MCF7 cells, the most widely used ER+/HER2− breast cancer cell line. Furthermore, our results underline the importance of using CTCs from patients with metastatic disease to study clinically-relevant resistance mechanisms, given they clearly exhibit different properties compared to established cell lines that are commonly used for such studies. Thus breast cancer CTCs are not only of great importance prognostically, they also represent a valuable tool for gaining new insights into endocrine resistance, late recurrence and metastatic progression, and for identifying drugs that can suppress these processes.

## 4. Materials and Methods

### 4.1. Cultivation of CTC-ITB-01 and Established MCF7 Cells

The establishment of CTC-ITB-01 cells in culture has been described previously [22]. They were cultured in RPMI supplemented with 10% fetal bovine serum (Takara Clontech, Heidelberg, Germany), 1% L-glutamine, 1% penicillin/streptomycin, 1% Transferrin Insulin Selenium (IST, Thermo Fisher Scientific, Dreieich, Germany), 50 ng/mL human research grade EGF (Miltenyi Biotech, Bergisch Gladbach, Germany), 10 ng/mL human basic fibroblast growth factor b-FGF (Miltenyi Biotech), 100 ng/mL Hydrocortisone (Sigma Aldrich, Taufkirchen, Germany) and 200 ng/mL cholera toxin (Sigma Aldrich). The human breast cancer cell line MCF7 and the human embryonic kidney cell line HEK293T were purchased from the American Type Culture Collection (ATCC, Manassas, VA, USA). Unless otherwise indicated, MCF7 cells were maintained in RPMI. HEK293T cells were maintained in DMEM. RPMI and DMEM were supplemented with 10% fetal bovine serum (Takara Clontech), 1% L-glutamine and 1% penicillin/streptomycin. Charcoal stripped fetal bovine serum was purchased from Thermo Fisher Scientific.

### 4.2. Viral Transduction

The packaging cell line HEK293T was used for generation of lentiviruses following standard calcium phosphate protocols. For all experiments, pooled transduced cell clones were used. 

### 4.3. Other Methods

Western blot analysis, FACS analysis and clonogenic assays were performed as previously described [61]. Quantitative RT-PCR was performed with Sybr Green (Invitrogen, Karlsruhe, Germany) according to the manufacturer’s instruction. 

### 4.4. HER2 CISH and Immunohistochemical Staining and Immunofluorescence

Immunohistochemistry (IHC) for the detection of HER2 expression was performed by using the Hercep *t*-test (Dako, Glostrup, Denmark). For the detection of ERα, PR, vimentin and E-cadherin, IHC was performed as described previously [61]. All IHC stainings were performed on an immunostainer (Autostainer +; Dako) according to the manufacturer’s instructions. Immunofluorescence (IF) for the detection of NFkB was performed by using an antibody against p65 and a secondary antibody conjugated to Alexa Fluor 488 (Dianova GmbH, Hamburg, Germany).

### 4.5. Cell Cycle Analysis

Cell cycle analysis was performed through direct DNA staining with propidium iodide (PI). Cells were resuspended in hypotonic fluorochrome solution (50 μg/mL PI in 0.1% sodium citrate plus 0.1% TritonX-100 (Sigma Aldrich, Darmstadt, Germany), then placed at 4 °C in the dark for a minimum of 1 h before flow cytometry analysis.

### 4.6. Statistical Analysis

Differences between experimental groups were assessed using the Student’s *t* test (Statistical Analysis System, Release 9.3, SAS Software, Heidelberg, Germany). *p* values of < 0.05 were considered significant.

4.7. shRNAs, Primer, Reagents and Antibodies

Carfilzomib was purchased from Amgen, Thousand Oaks, CA, USA or from Selleckchem, Munich, Germany. AZD9496 and Lapatinib Ditosylat were purchased from Selleckchem. Fulvestrant and human TNFα were purchased from Sigma Aldrich. Antibodies for detection of ERα (D-12): sc-8005, Progesterone receptor for WB: ab63605 was purchased from abcam (Cambridge, UK). Ki67 for IHC: ab15580 was purchased from abcam. Progesterone receptor for IHC (clone PgR 636), vimentin (clone V9) and E-cadherin (clone NCH-38) were purchased from Agilent (Santa Clara, CA, USA). β-actin (C-4): sc-47778, Akt (H-136): sc-8312 were purchased from Santa Cruz Biotechnology (Heidelberg, Germany). Antibodies for detection of HER2 (#2242), p-Akt^S473^ (#9271), PARP1 (#9532) and caspase-7 (#9492) and p65 (#8242) were purchased from Cell Signaling Technology (Heidelberg, Germany). shRNA against human ERα, human HER2 and human FOXM1 and non-targeted shRNA were purchased from Sigma Aldrich. ER alpha shRNA1: TRCN0000003298, ER alpha shRNA2:TRCN0000003300, HER2 shRNA1: TRCN0000332953, HER2 shRNA2: TRCN 0000039878, FOXM1 shRNA1:TRCN0000015544, FOXM1 shRNA2:TRC N000005546. Primer ER alpha fw: attggccagtaccaatgacaaggg, ER alpha rev: tatcaatggtgcactggttggtgg; FOXM1 fw: acctgcagcta gggatgtgaatct, FOXM1 rev: aagccactggatgttggataggct, HER2 fw: agcggtgtgaaacctgacc, HER2 rev: ttgatgaggatcccaaagacc; RibPO fw: agacaatgtgggctccaagcagat, RibPO rev: gcatcatggtgttcttgcccatca.

## 5. Conclusions

Mechanisms of acquired endocrine resistance and late recurrence in patients with ER+/HER2− breast cancer are complex and not fully understood. Authentic in vitro or in vivo models derived from patients that allow acquired resistance to endocrine treatment to be investigated mechanistically are rare. Here, we used CTCs from patients with acquired endocrine resistance to investigate mechanisms of endocrine resistance. We found that CTCs are resistant to ERα inhibition, and that HER2 is required for sustained FOXM1 expression even in ER+ breast cancer cells that do not have HER2 amplification or activating HER2 mutations. Our results point to a switch from ERα-dependent to HER2-dependent and ERα-independent expression of FOXM1 when patients progress under endocrine treatment. Our results also show that inhibition of the NFkB signaling pathway in CTCs suppresses HER2 and FOXM1 expression and causes cell death. These findings suggest that targeting of NFkB and FOXM1 might be an efficient therapeutic approach to prevent late recurrence and to treat endocrine resistance. We conclude that CTCs are of importance for functional studies to investigate mechanisms of acquired endocrine resistance as well as for the testing of potential drugs to target these mechanisms.

## Data Availability

The data presented in this study are available on request from the corresponding author.

## Supplementary Materials

The following are available online at https://www.mdpi.com/article/10.3390/cancers13081810/s1, Figure S1. CTCs were derived from a patient with bi-lateral metastatic breast cancer, Figure S2. (A) + (B) Knockdown of FOXM1 displays differences between CTC-ITB-01 and MCF7 cells, Figure S3. (A) + (B) CTC-ITB-01 and MCF7 cells were grown on cell culture plates or cultured in suspension, Figure S4. CTC-ITB-01 cells were cultured in suspension in the absence or presence of 30 µM carfilzomib, Figure S5. (A) + (B) CTC-ITB-01 and MCF7 cells were serum starved overnight, than treated with TNFα + JSH-23 or with TNFα alone, and subsequently fixed and stained with an NFkB p65 antibody 20 min after TNFα treatment, Figure S6. (A) + (B) CTC-ITB-01 and MCF7 cells were cultured in the presence or absence of JSH-23. mRNA was harvested and FOXM1 transcripts were quantified by qPCR, Figure S7. Original Uncropped Western Blot Images.
