# Supplementary material for: Functional Characterization of Circulating Tumor Cells (CTCs) from Metastatic ER+/HER2− Breast Cancer Reveals Dependence on HER2 and FOXM1 for Endocrine Therapy Resistance and Tumor Cell Survival: Implications for Treatment of ER+/HER2− Breast Cancer
Source: Cancers (Basel). 2021 Apr 10;13(8):1810. doi: 10.3390/cancers13081810 (PMC8070196; doi:10.3390/cancers13081810)
Supplement: Supplementary file 1 [file cancers-13-01810-s001.pdf]

Supplementary Materials: Functional Characterization of Circulating Tumor Cells (CTCs) from Metastatic ER+/HER2− Breast Cancer Reveals Dependence on HER2 and FOXM1 for Endocrine Therapy Resistance and Tumor Cell Survival: Implications for Treatment of ER+/HER2− Breast Cancer

Sven Roßwag, Cristina L. Cotarelo, Klaus Pantel, Sabine Riethdorf, Jonathan P. Sleeman, Marcus Schmidt and Sonja Thaler

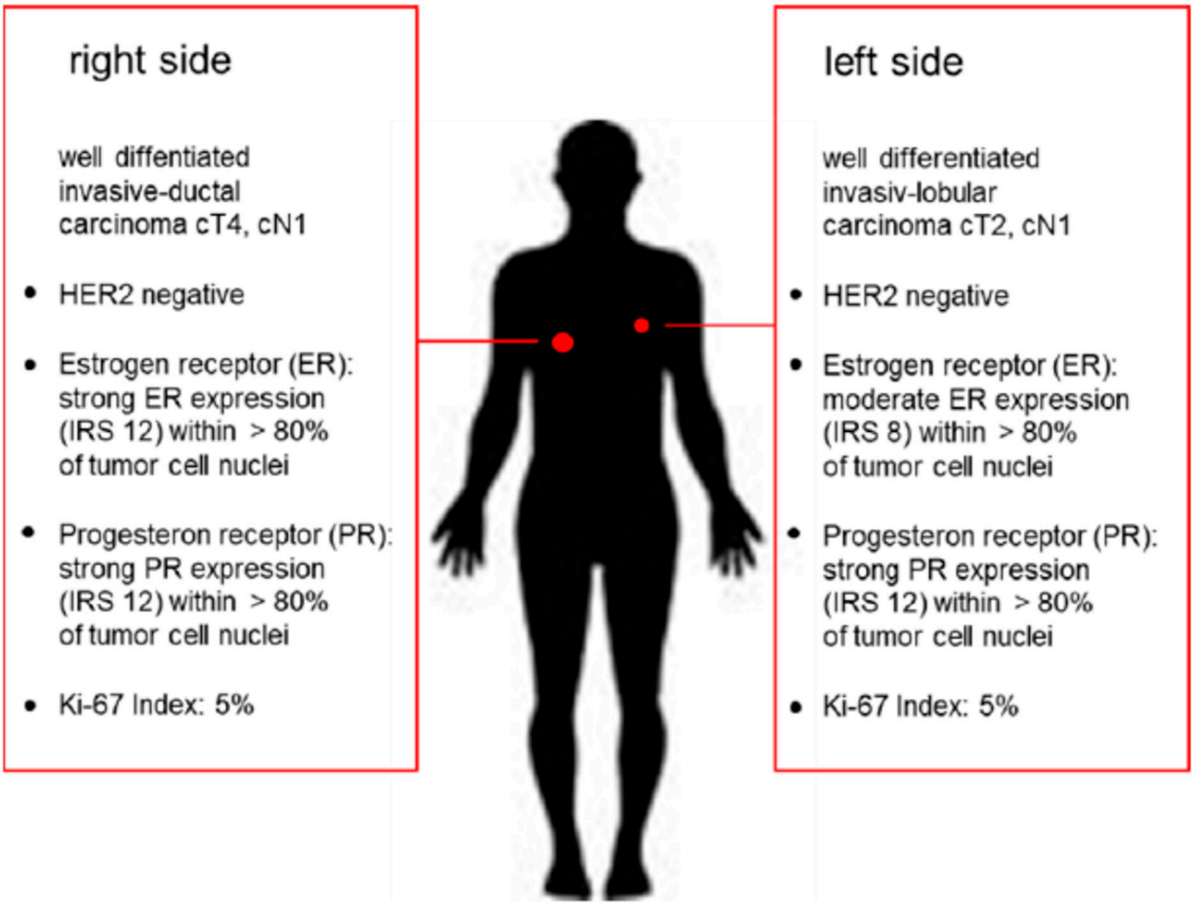

Figure S1. CTCs were derived from a patient with bi-lateral metastatic breast cancer. The patient was originally diagnosed with an invasive ductal carcinoma and an invasive lobular carcinoma. Characteristics of the tumors are given in the Figure.

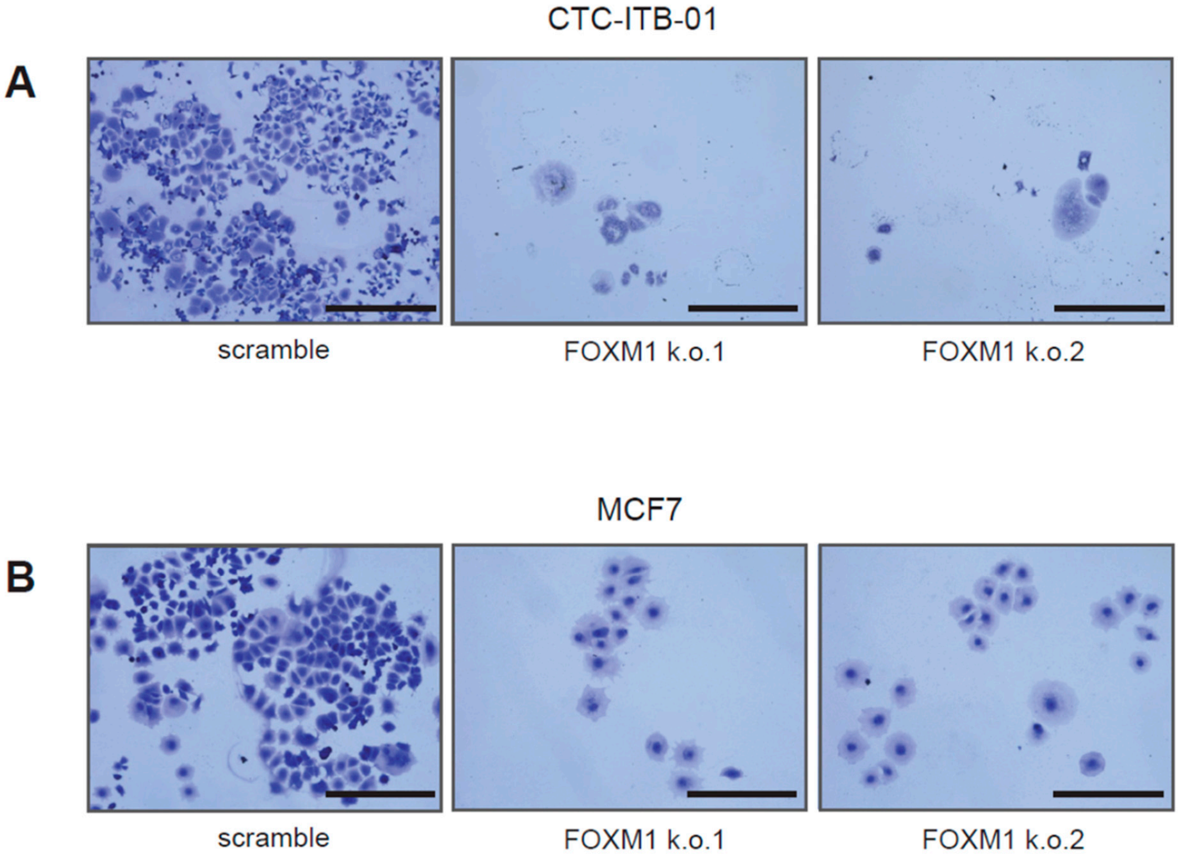

Figure S2. (**A**) + (**B**) Knockdown of FOXM1 displays differences between CTC-ITB-01 and MCF7 cells. (**B**) Knockdown of FOXM1 in MCF7 cells causes less reduction of colonies as observed within CTC-ITB-01 and induction of senescence as monitored by typical morphological changes. (**A**) In CTC-ITB-01 cells, we observed less cells with senescence-like morphology and cell debris indicating that FOXM1 knockdown causes mainly induction of senescence within MCF7 and more cell death within CTC-ITB-01 cells. Bar 400 µm.

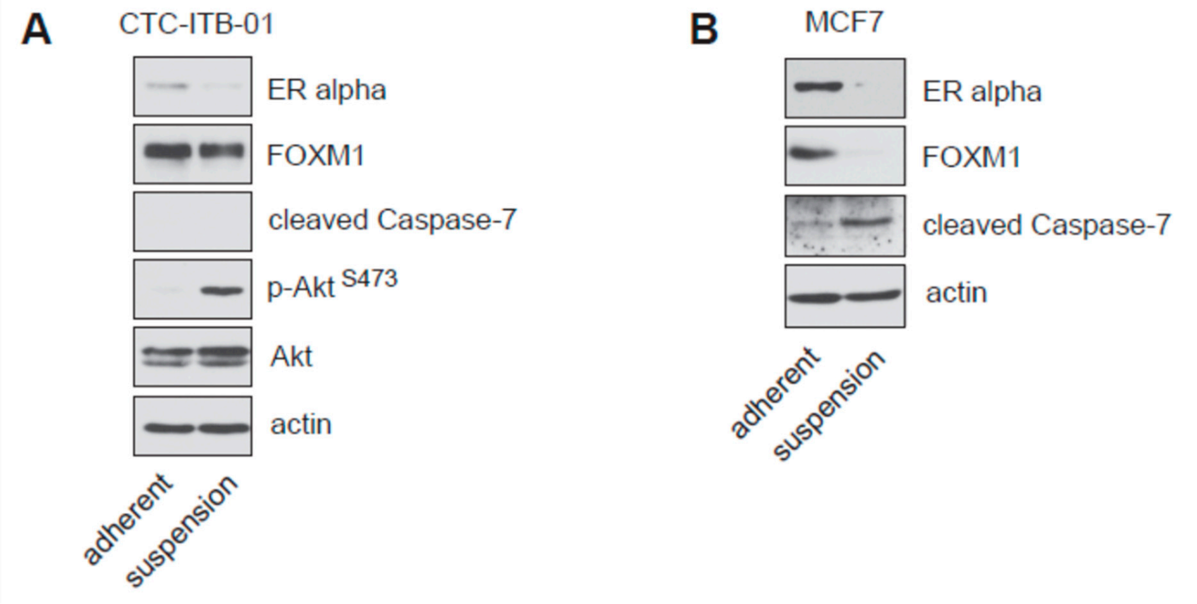

Figure S3. (**A**) + (**B**) CTC-ITB-01 and MCF7 cells were grown on cell culture plates or cultured in suspension. 24 hours after seeding cells on tissue culture plates or taking cells in suspension CTC-ITB-01 and MCF7 cells were harvested and lysates were analysed by western blotting and probed by using the indicated antibodies.

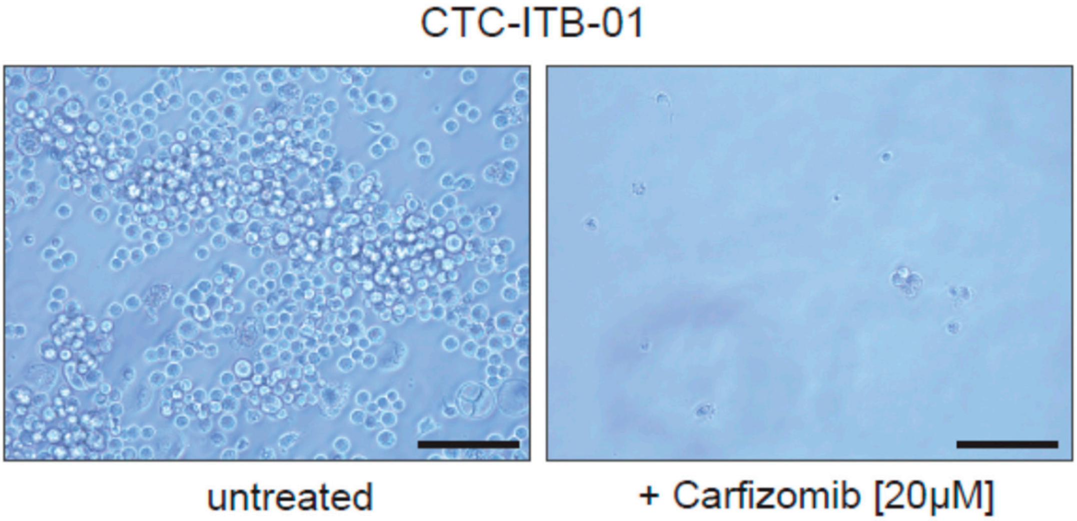

Figure S4. CTC-ITB-01 cells were cultured in suspension in the absence or presence of 30 μM carfilzomib. After five days carfilzomib treatment lead to induction of apoptotic cell death.

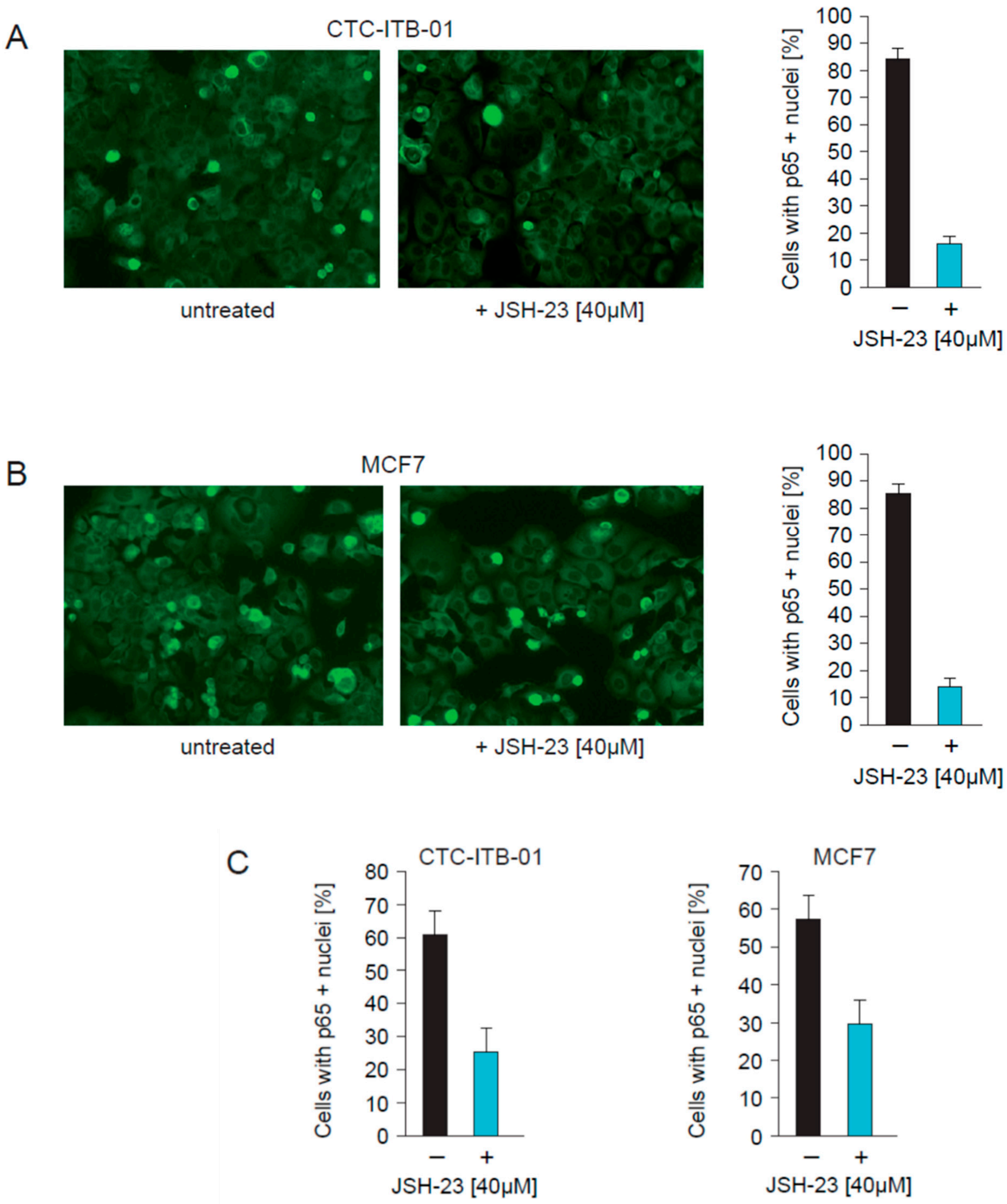

Figure S5. (**A**) + (**B**) CTC-ITB-01 and MCF7 cells were serum starved overnight, than treated with TNFα + JSH-23 or with TNFα alone, and subsequently fixed and stained with an NFkB p65 antibody 20 min after TNFα treatment. In the presence of JSH-23 a strong reduction of cells with p65+ nuclei in the CTC-ITB-01 cells and in the MCF7 cells was observed. (**C**) After establishing the NF-kB p65 staining in this way, we cultured CTC-ITB-01 and MCF7 cells with JSH-23 as described in Figure 6D in the manuscript. Afterwards we repeated the NF-kB p65 staining and quantified the percentage of cells with p65+ nuclei. Again, we observed strong reduction of cells with p65+ nuclei (**C**) CTC-ITB-01 left panel, MCF7 cells right panel.

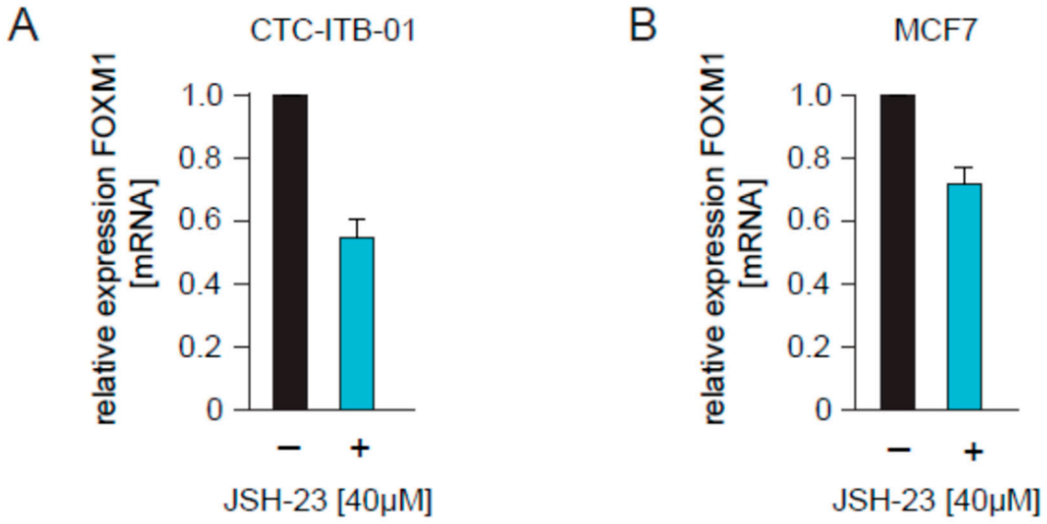

Figure S6. (**A**) + (**B**) CTC-ITB-01 and MCF7 cells were cultured in the presence or absence of JSH-23. mRNA was harvested and FOXM1 transcripts were quantified by qPCR.

Figure 1B

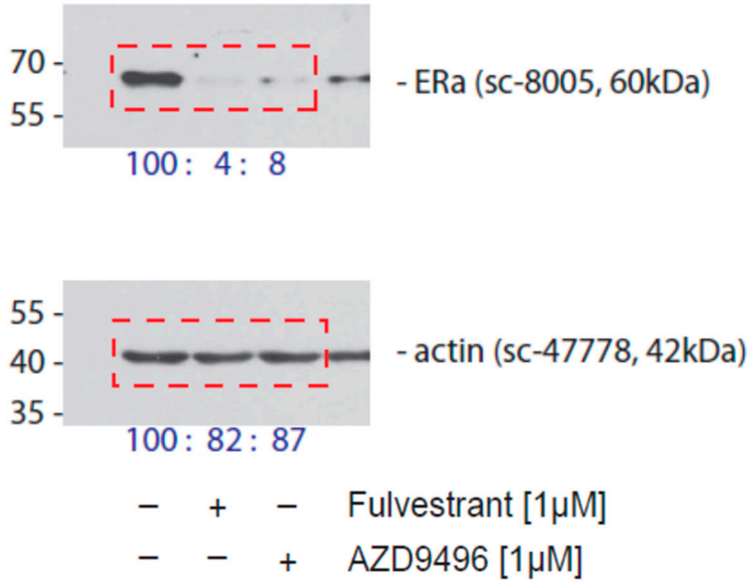
70 -
55 -
- ERa (sc-8005, 60kDa)
100 : 4 : 8
55 -
40 -
35 -
- actin (sc-47778, 42kDa)
100 : 82 : 87
− + − Fulvestrant [1μM]
− − + AZD9496 [1μM]

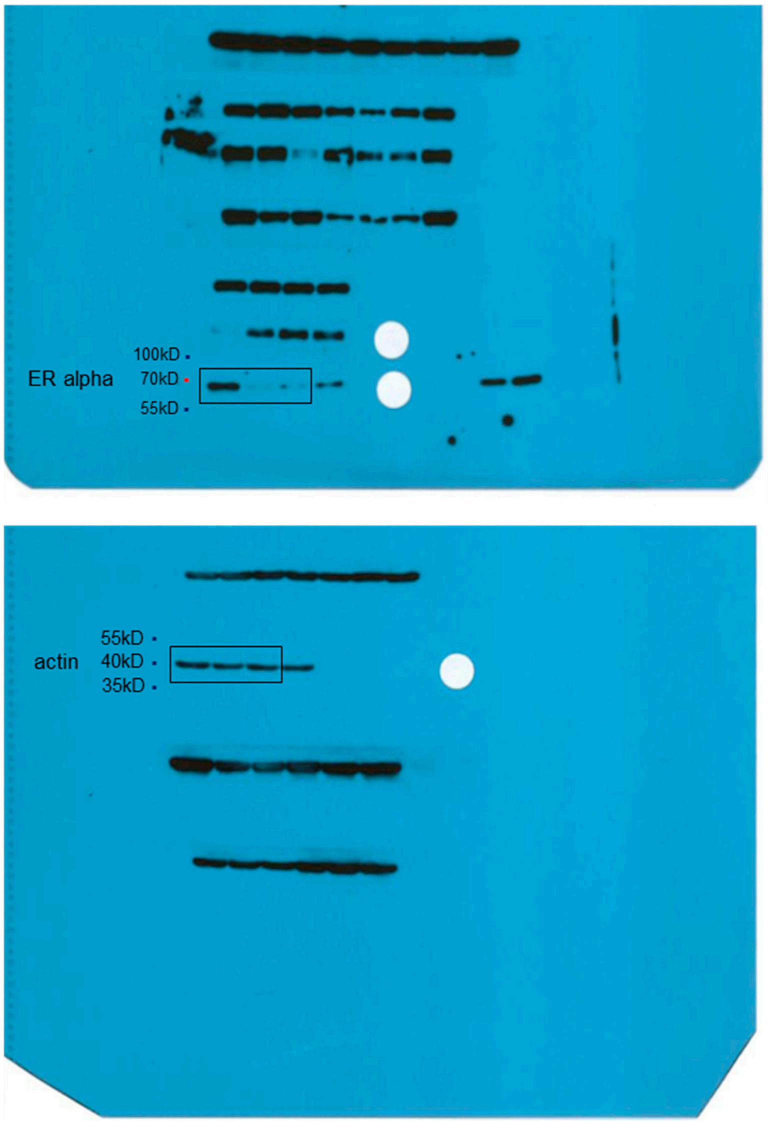
ER alpha
100kD
70kD
55kD
actin
55kD
40kD
35kD

Figure 1C

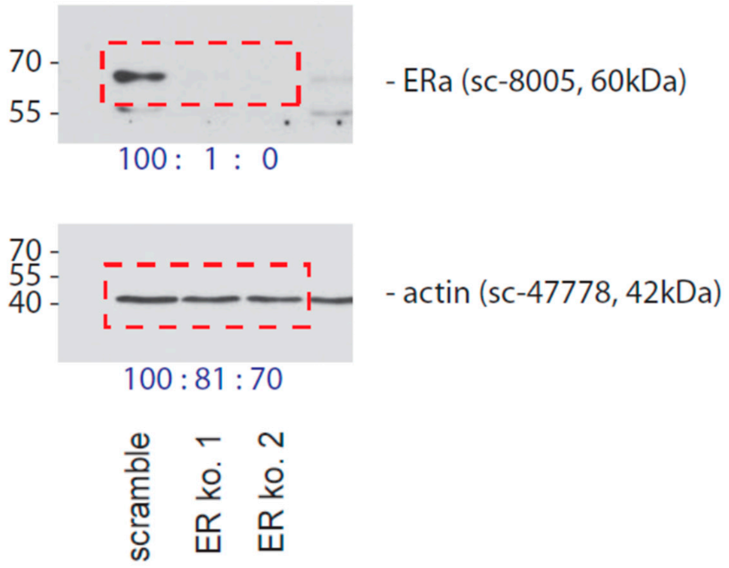
70 -
55 -
100 : 1 : 0
- ERa (sc-8005, 60kDa)
70 -
55 -
40 -
100 : 81 : 70
- actin (sc-47778, 42kDa)
scramble
ER ko. 1
ER ko. 2

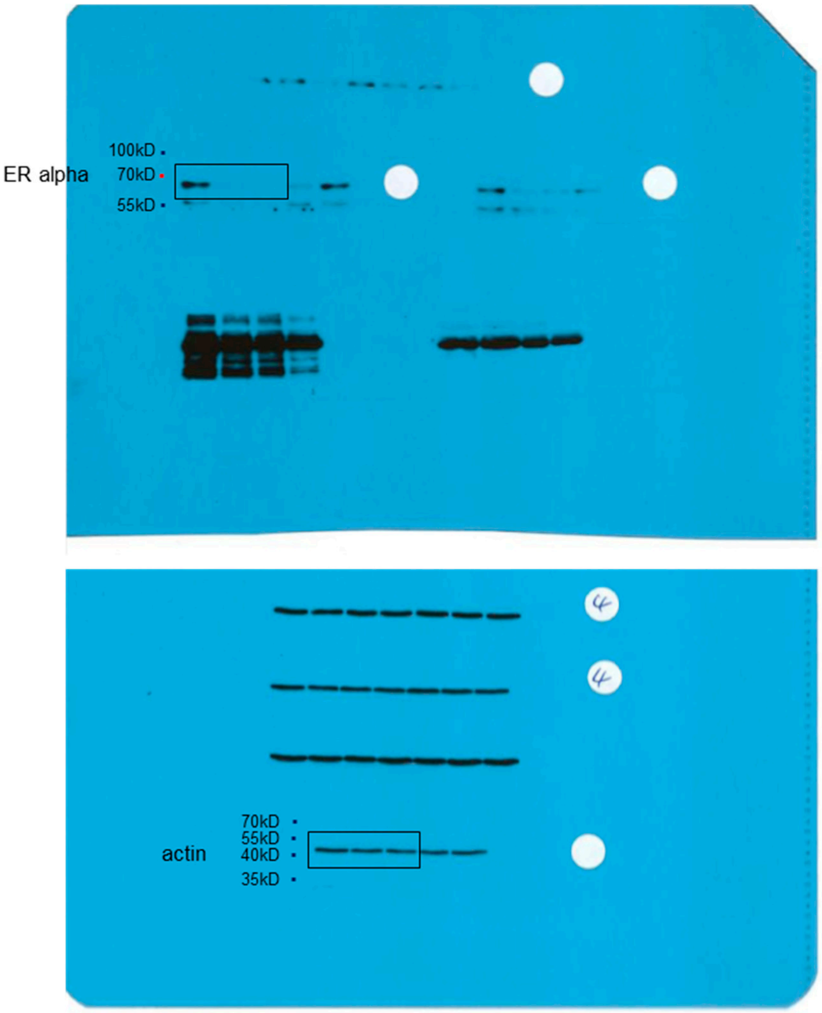
ER alpha
100kD
70kD
55kD
actin
70kD
55kD
40kD
35kD

Figure 1D

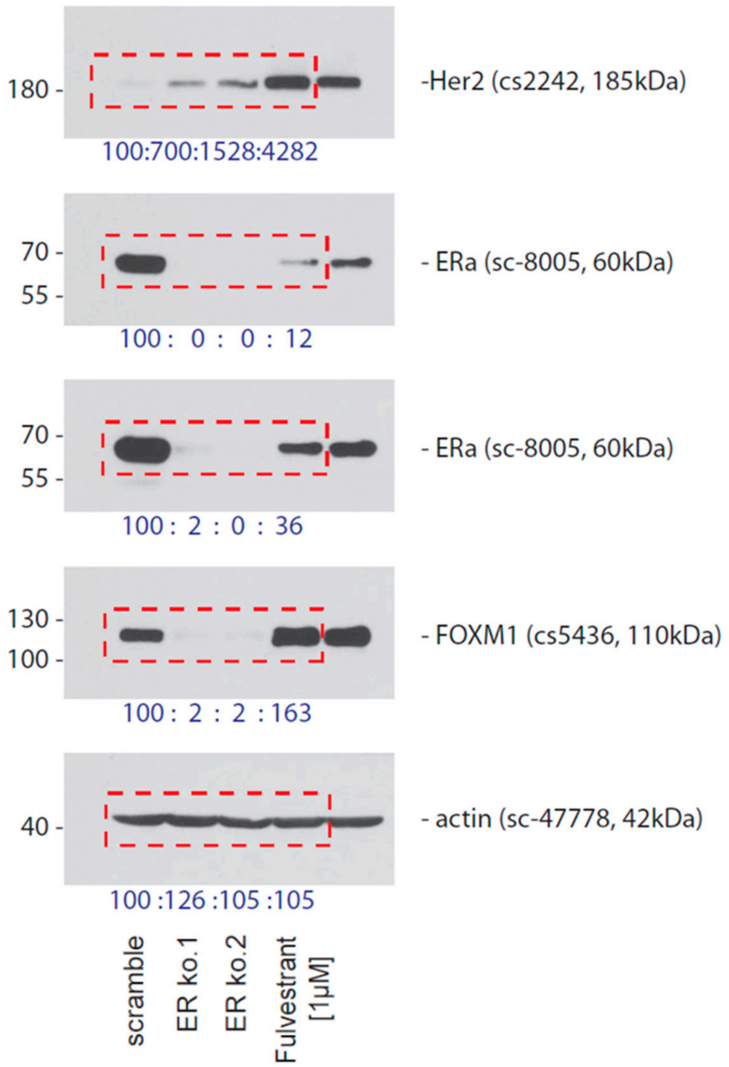
180 -
-Her2 (cs2242, 185kDa)
100:700:1528:4282
70 -
55 -
- ERa (sc-8005, 60kDa)
100 : 0 : 0 : 12
70 -
55 -
- ERa (sc-8005, 60kDa)
100 : 2 : 0 : 36
130 -
100 -
- FOXM1 (cs5436, 110kDa)
100 : 2 : 2 : 163
40 -
- actin (sc-47778, 42kDa)
100 :126 :105 :105
scramble
ER ko.1
ER ko.2
Fulvestrant [1µM]

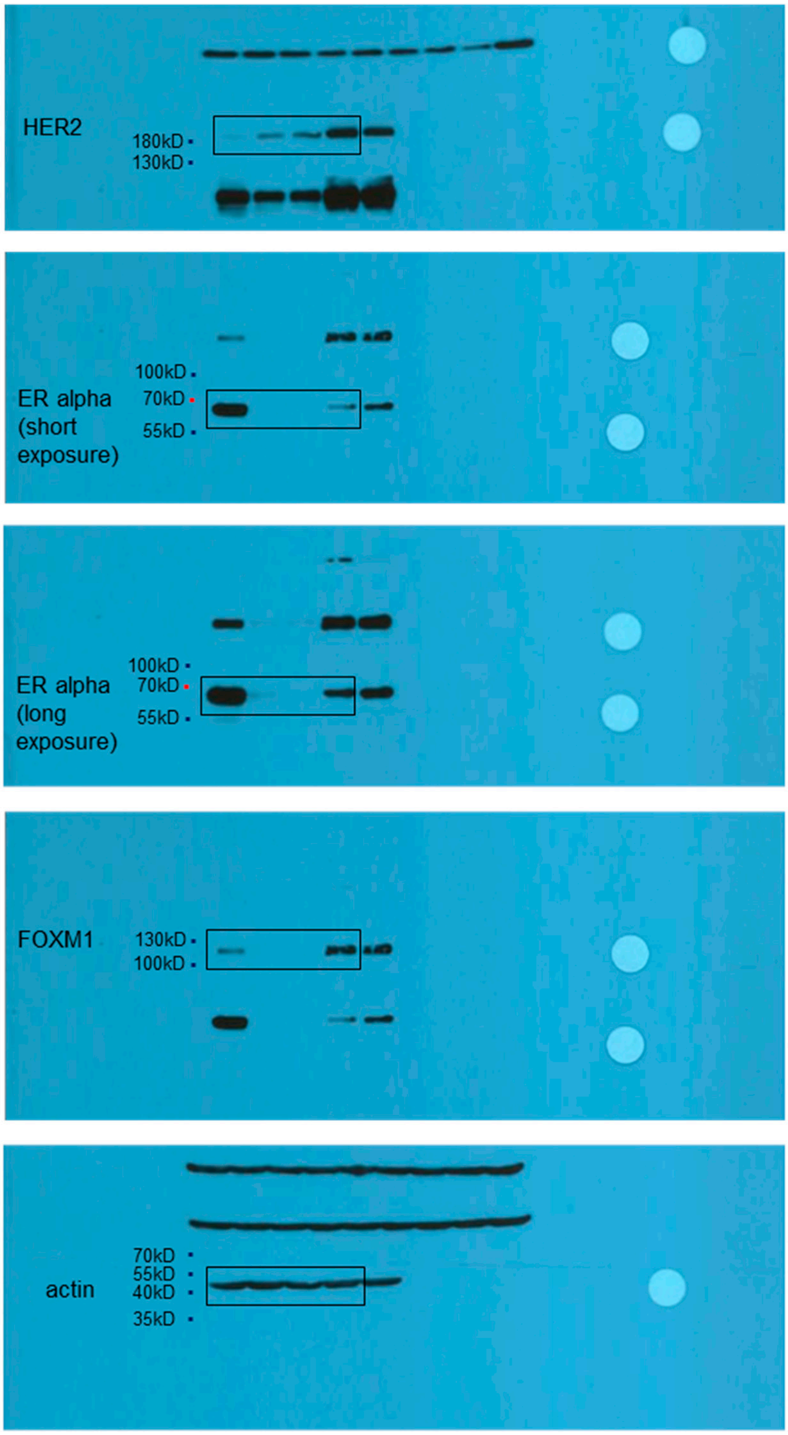
HER2
180kD
130kD
100kD
ER alpha (short exposure)
70kD
55kD
100kD
ER alpha (long exposure)
70kD
55kD
FOXM1
130kD
100kD
actin
70kD
55kD
40kD
35kD

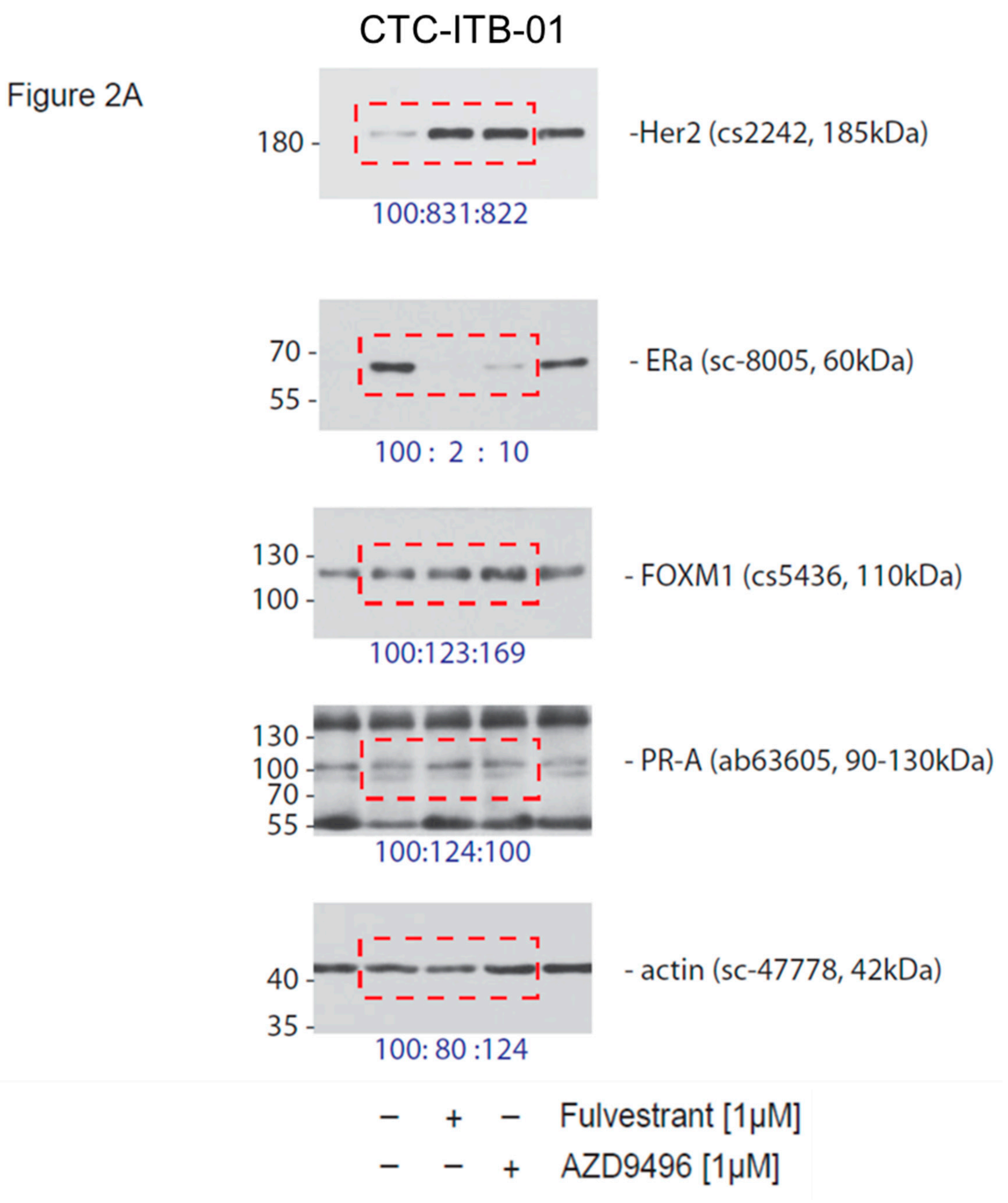
CTC-ITB-01
Figure 2A
180 -
-Her2 (cs2242, 185kDa)
100:831:822
70 -
55 -
- ERa (sc-8005, 60kDa)
100 : 2 : 10
130 -
100 -
- FOXM1 (cs5436, 110kDa)
100:123:169
130 -
100 -
70 -
55 -
- PR-A (ab63605, 90-130kDa)
100:124:100
40 -
35 -
- actin (sc-47778, 42kDa)
100: 80 :124
– + – Fulvestrant [1μM]
– – + AZD9496 [1μM]

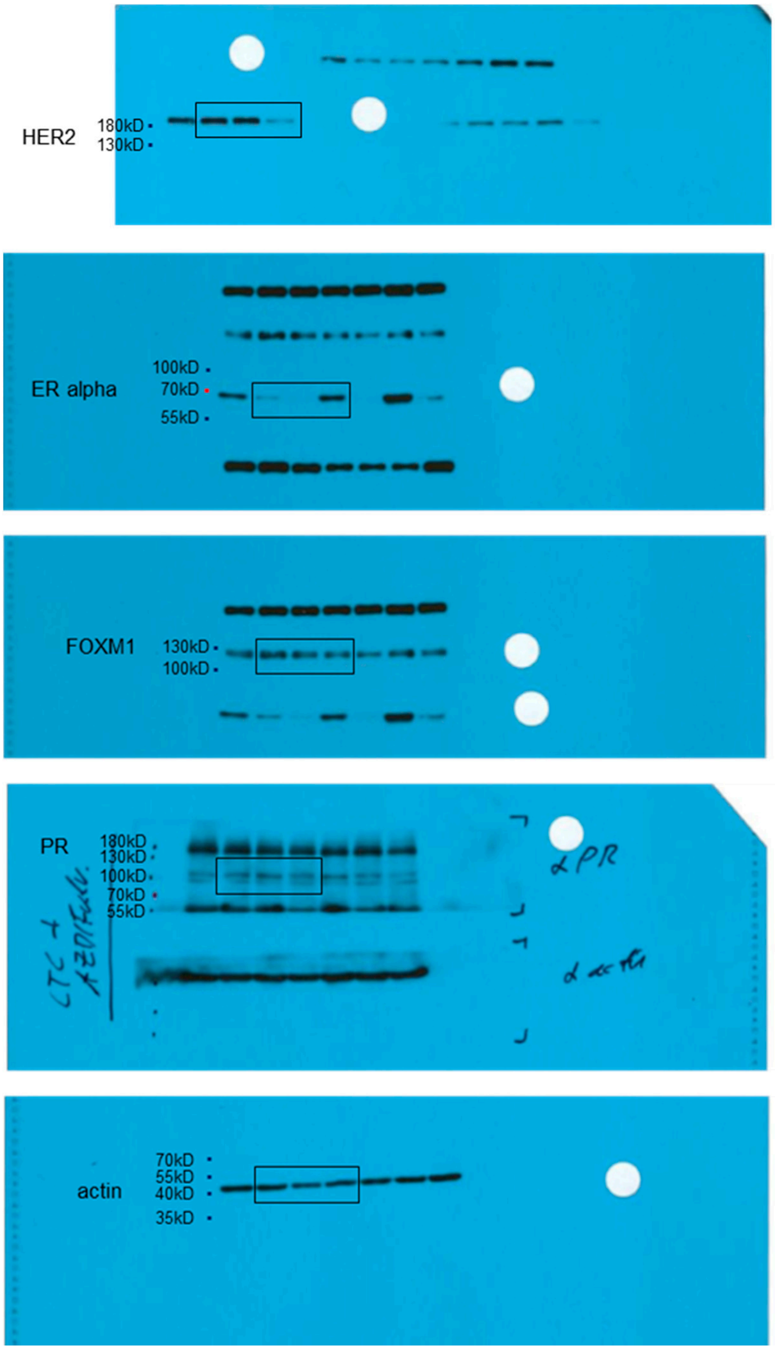
HER2
180kD
130kD
ER alpha
100kD
70kD
55kD
FOXM1
130kD
100kD
PR
180kD
130kD
100kD
70kD
55kD
actin
70kD
55kD
40kD
35kD

Figure 2C

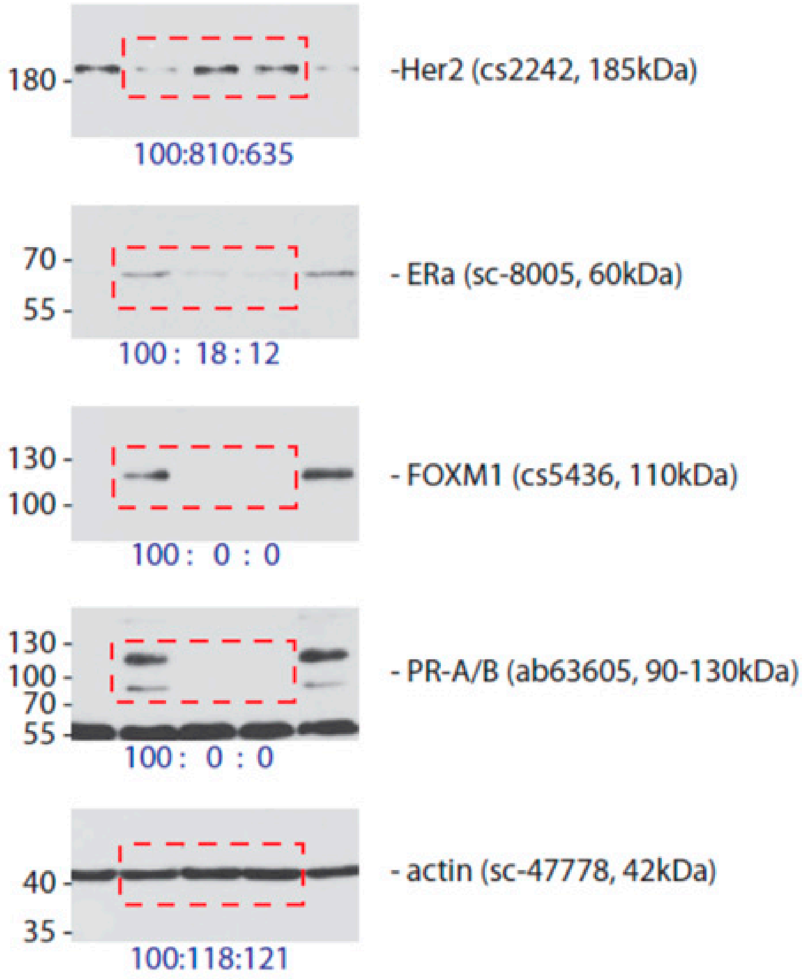
180 -
-Her2 (cs2242, 185kDa)
100:810:635
70 -
55 -
- ERa (sc-8005, 60kDa)
100 : 18 : 12
130 -
100 -
- FOXM1 (cs5436, 110kDa)
100 : 0 : 0
130 -
100 -
70 -
55 -
- PR-A/B (ab63605, 90-130kDa)
100 : 0 : 0
40 -
35 -
- actin (sc-47778, 42kDa)
100:118:121

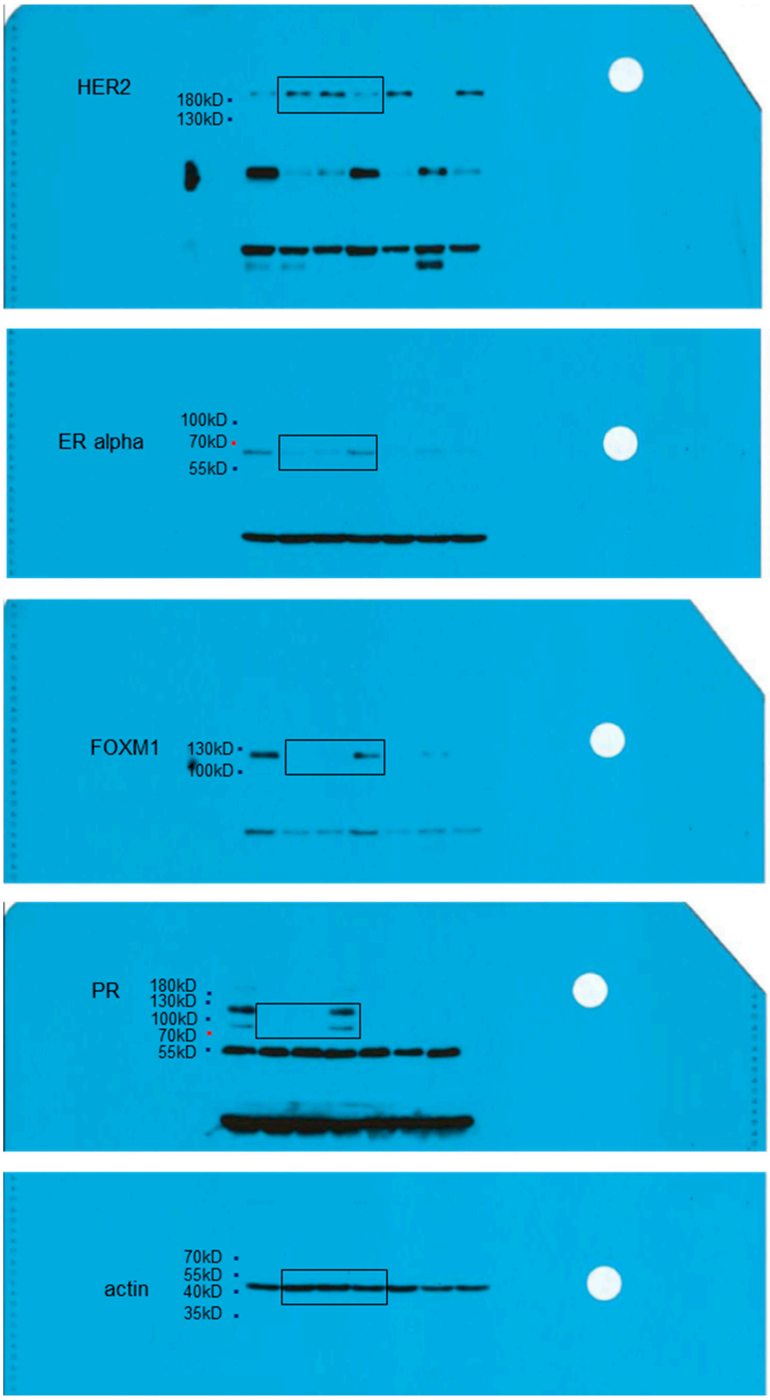
HER2
180kD
130kD
ER alpha
100kD
70kD
55kD
FOXM1
130kD
100kD
PR
180kD
130kD
100kD
70kD
55kD
actin
70kD
55kD
40kD
35kD

Figure 3A

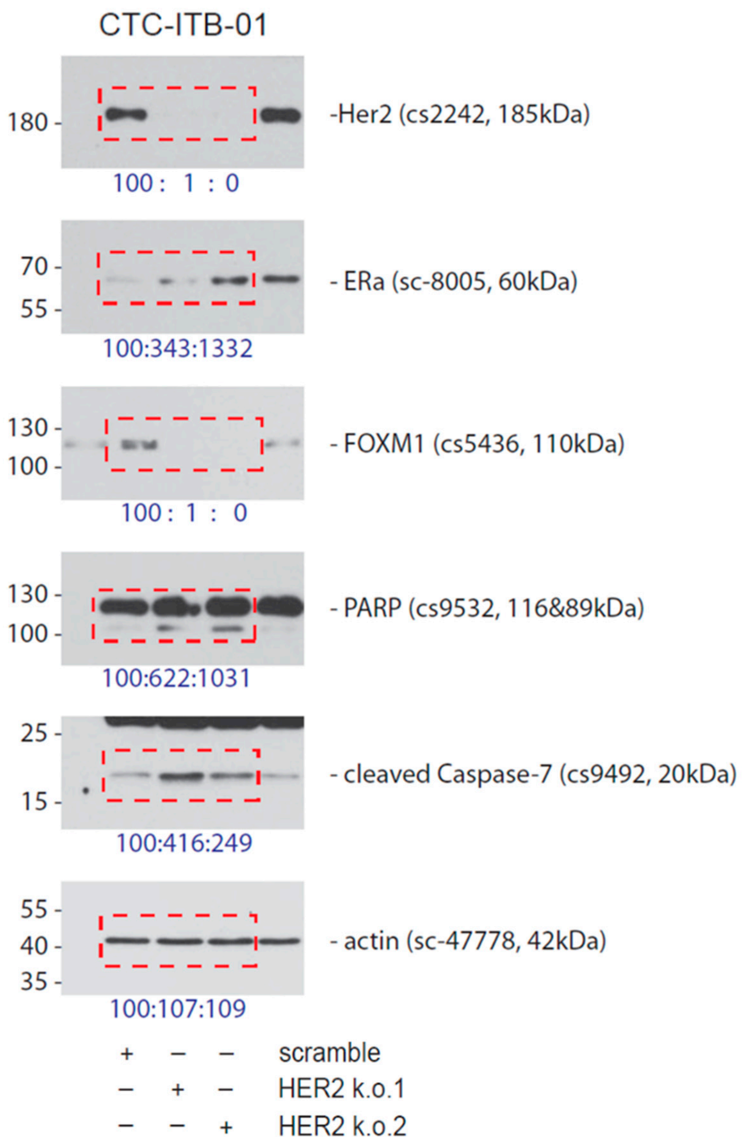
CTC-ITB-01
180 -
-Her2 (cs2242, 185kDa)
100 : 1 : 0
70 -
55 -
- ERa (sc-8005, 60kDa)
100:343:1332
130 -
100 -
- FOXM1 (cs5436, 110kDa)
100 : 1 : 0
130 -
100 -
- PARP (cs9532, 116&89kDa)
100:622:1031
25 -
15 -
- cleaved Caspase-7 (cs9492, 20kDa)
100:416:249
55 -
40 -
35 -
- actin (sc-47778, 42kDa)
100:107:109
+ – – scramble
– + – HER2 k.o.1
– – + HER2 k.o.2

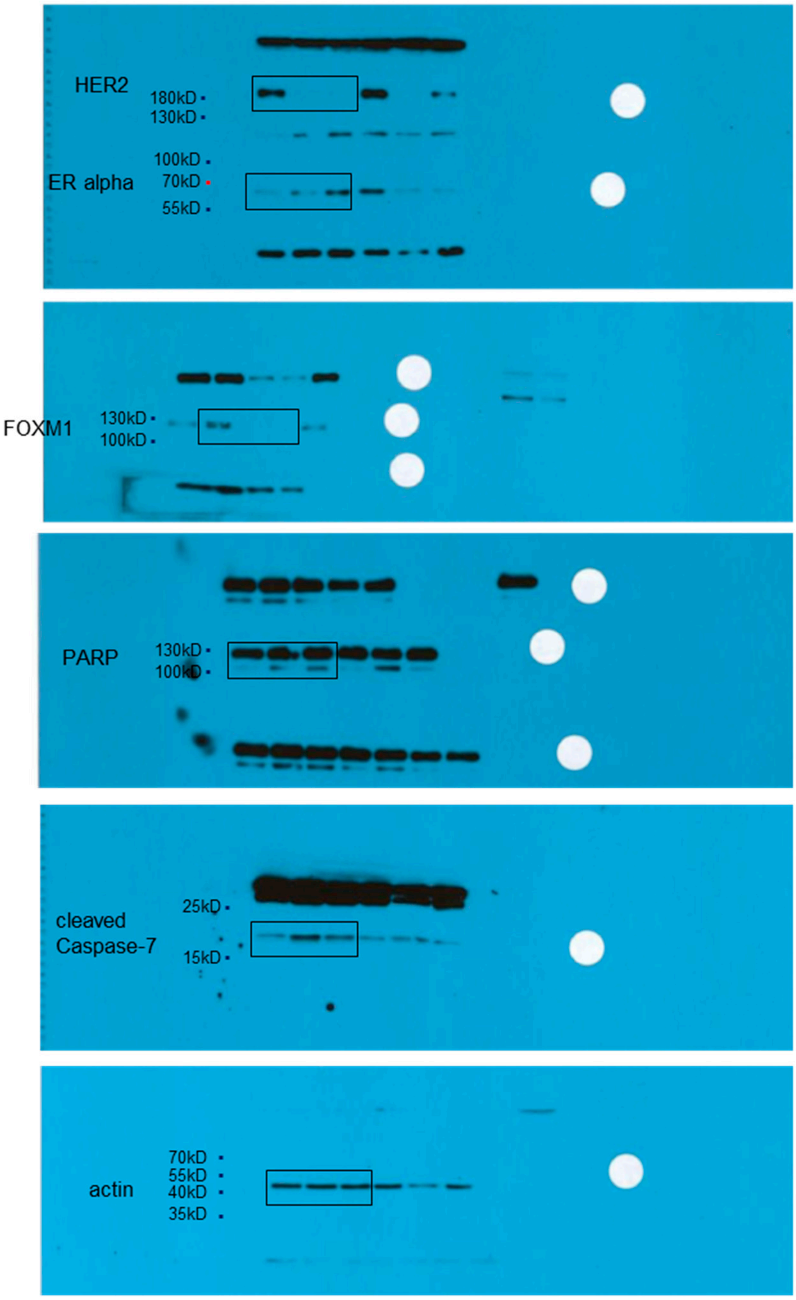
HER2
180kD
130kD
100kD
ER alpha
70kD
55kD
FOXM1
130kD
100kD
PARP
130kD
100kD
cleaved
Caspase-7
25kD
15kD
actin
70kD
55kD
40kD
35kD

Figure 3B

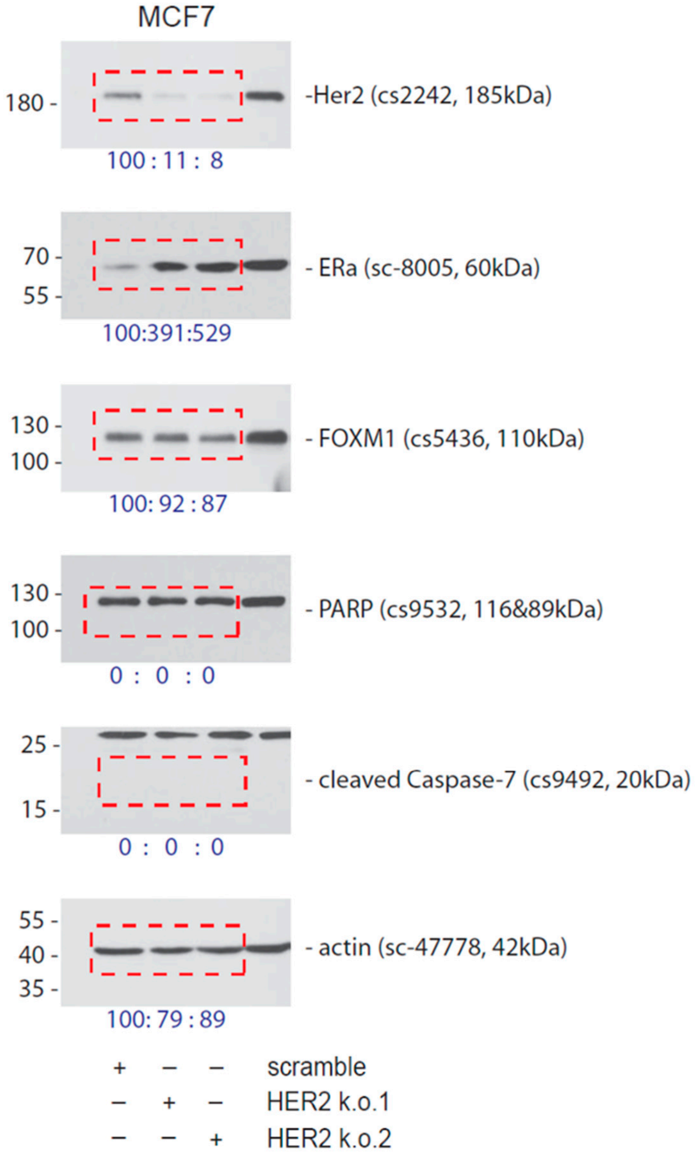
MCF7
180 -
-Her2 (cs2242, 185kDa)
100 : 11 : 8
70 -
55 -
- ERa (sc-8005, 60kDa)
100:391:529
130 -
100 -
- FOXM1 (cs5436, 110kDa)
100: 92 : 87
130 -
100 -
- PARP (cs9532, 116&89kDa)
0 : 0 : 0
25 -
15 -
- cleaved Caspase-7 (cs9492, 20kDa)
0 : 0 : 0
55 -
40 -
35 -
- actin (sc-47778, 42kDa)
100: 79 : 89
+ − − scramble
− + − HER2 k.o.1
− − + HER2 k.o.2

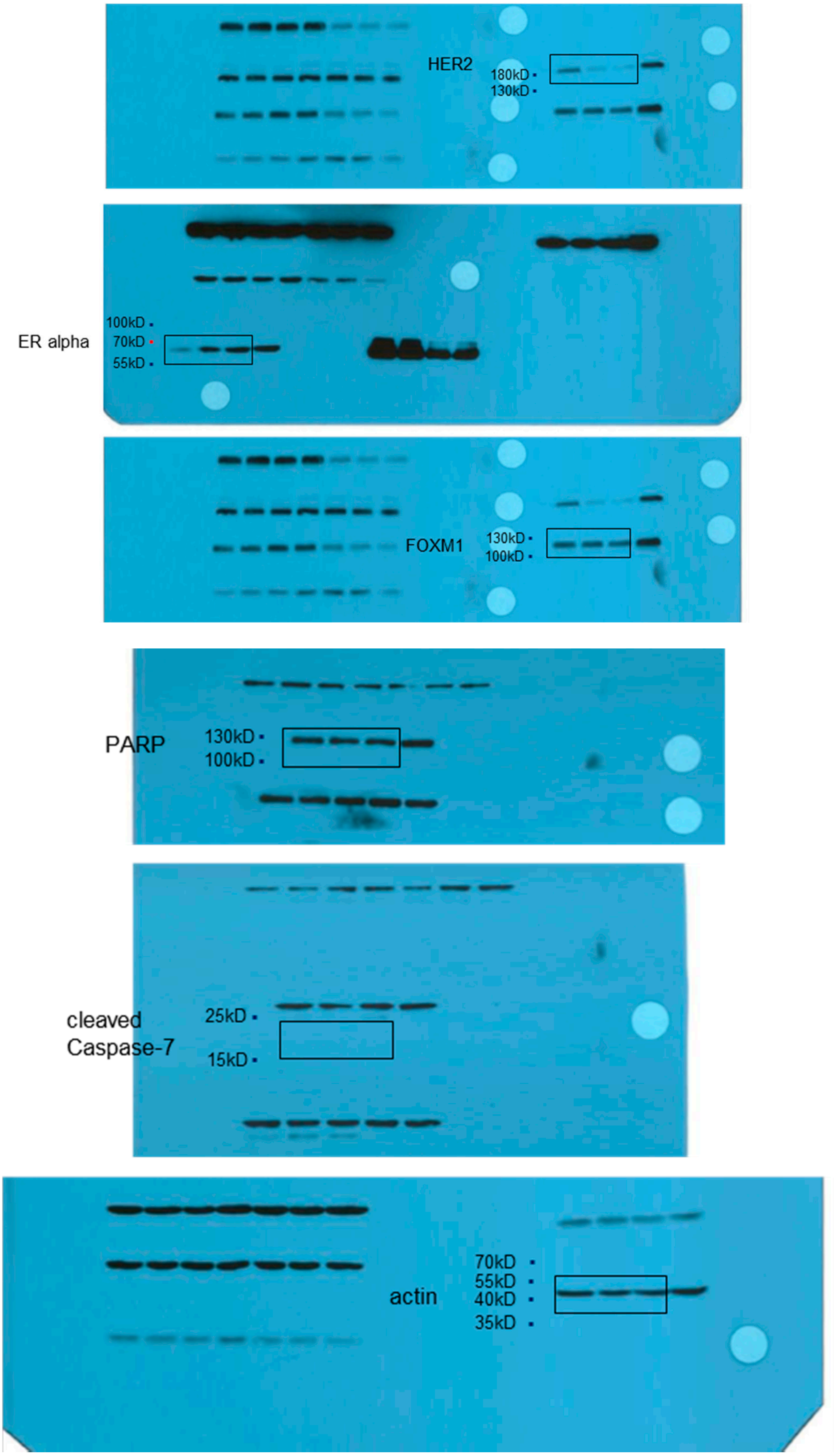
HER2
180kD
130kD
ER alpha
100kD
70kD
55kD
FOXM1
130kD
100kD
PARP
130kD
100kD
cleaved
Caspase-7
25kD
15kD
actin
70kD
55kD
40kD
35kD

Figure 3C

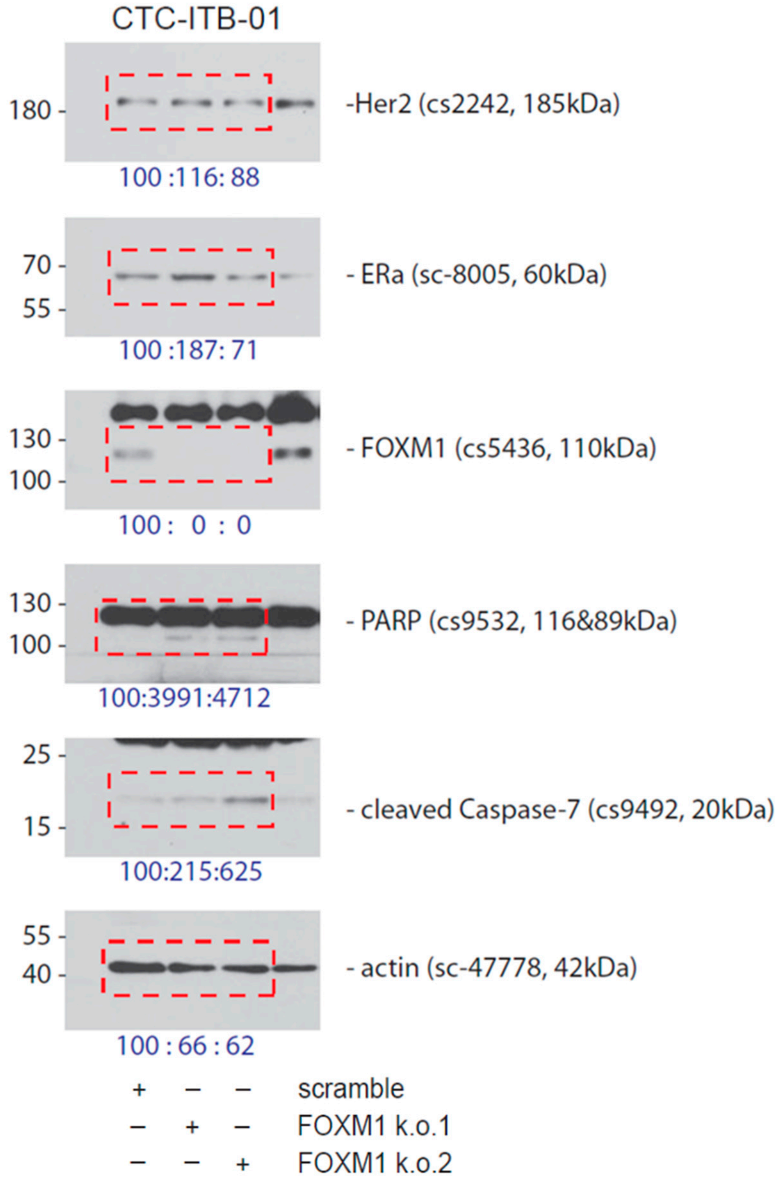
CTC-ITB-01
180 -
-Her2 (cs2242, 185kDa)
100 :116: 88
70 -
55 -
- ERa (sc-8005, 60kDa)
100 :187: 71
130 -
100 -
- FOXM1 (cs5436, 110kDa)
100 : 0 : 0
130 -
100 -
- PARP (cs9532, 116&89kDa)
100:3991:4712
25 -
15 -
- cleaved Caspase-7 (cs9492, 20kDa)
100:215:625
55 -
40 -
- actin (sc-47778, 42kDa)
100 : 66 : 62
+ – – scramble
– + – FOXM1 k.o.1
– – + FOXM1 k.o.2

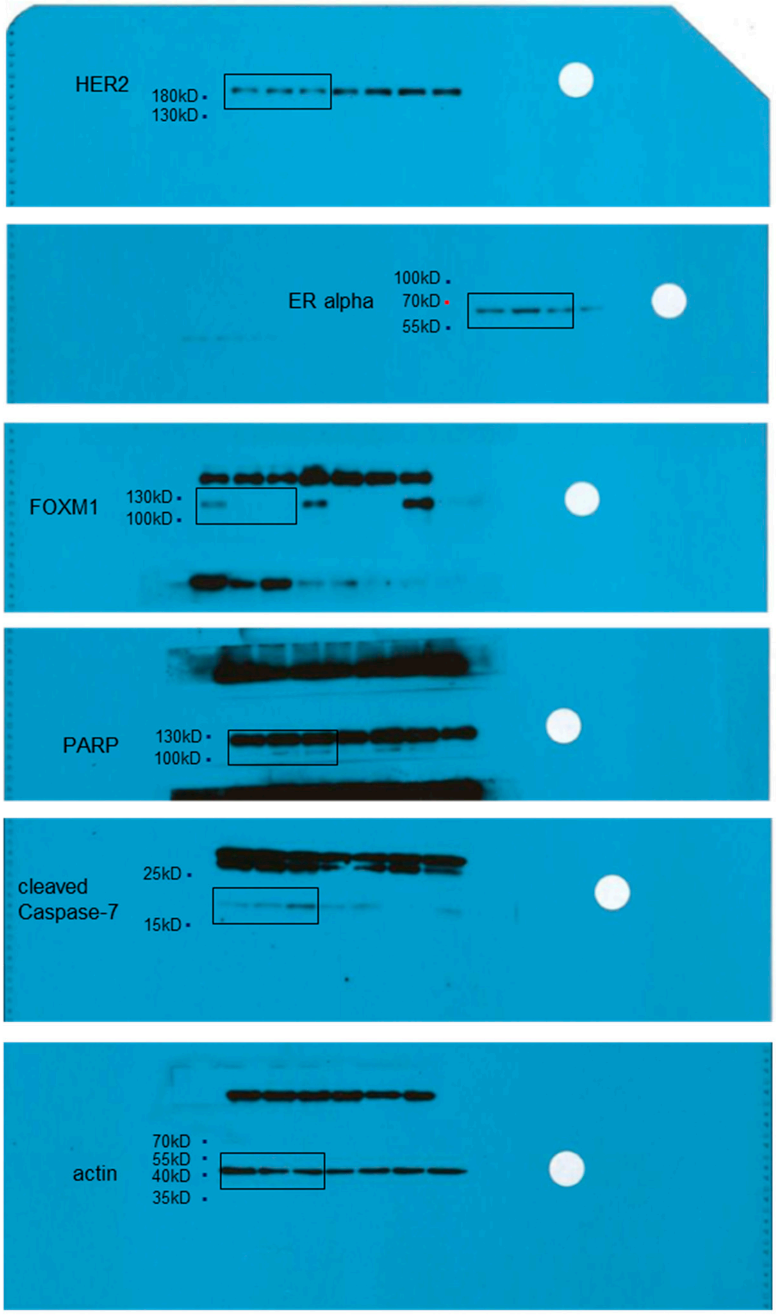

HER2
180kD
130kD
ER alpha
100kD
70kD
55kD
FOXM1
130kD
100kD
PARP
130kD
100kD
cleaved
Caspase-7
25kD
15kD
actin
70kD
55kD
40kD
35kD

Figure 3D

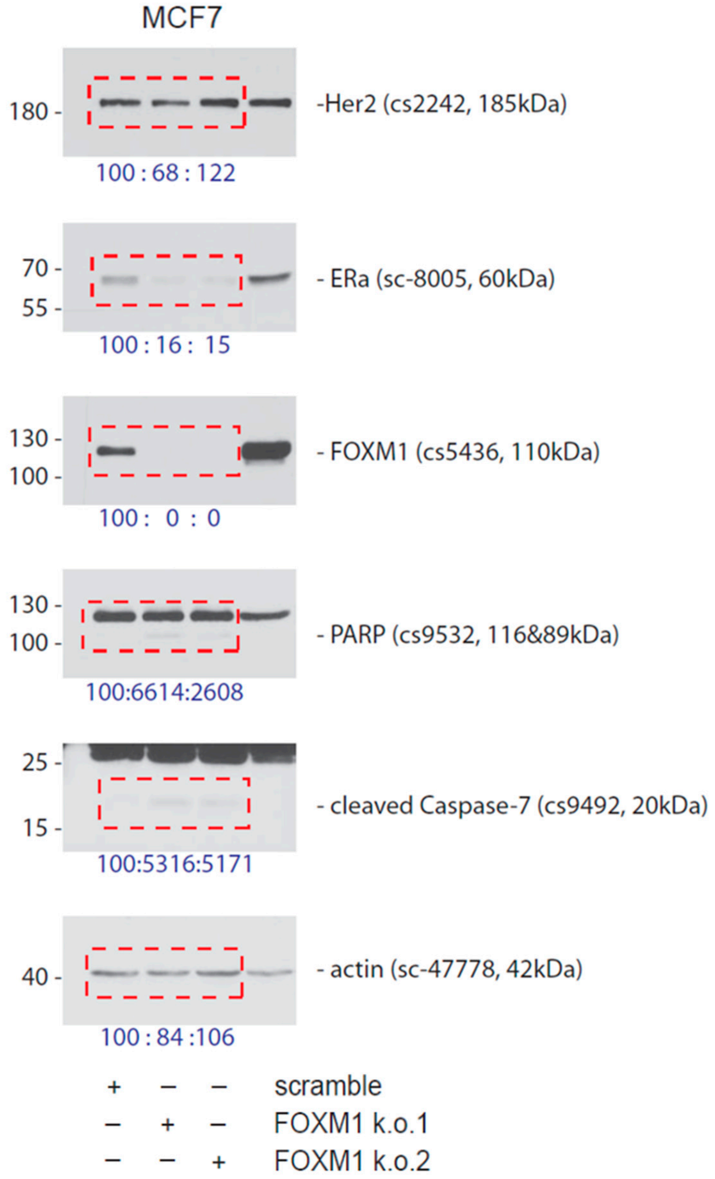
MCF7
180 -
-Her2 (cs2242, 185kDa)
100 : 68 : 122
70 -
55 -
- ERa (sc-8005, 60kDa)
100 : 16 : 15
130 -
100 -
- FOXM1 (cs5436, 110kDa)
100 : 0 : 0
130 -
100 -
- PARP (cs9532, 116&89kDa)
100:6614:2608
25 -
15 -
- cleaved Caspase-7 (cs9492, 20kDa)
100:5316:5171
40 -
- actin (sc-47778, 42kDa)
100 : 84 :106
+ − − scramble
− + − FOXM1 k.o.1
− − + FOXM1 k.o.2

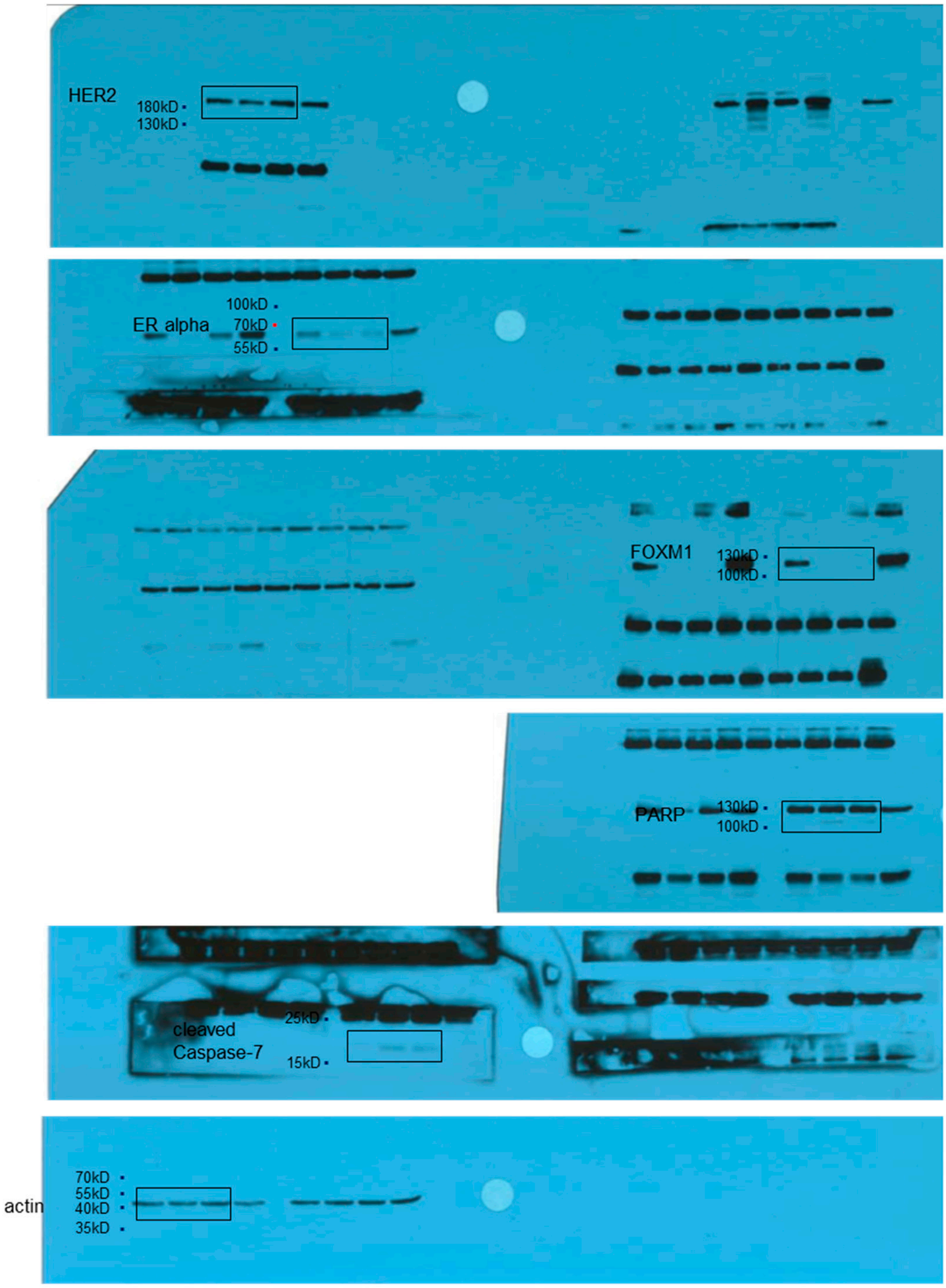
HER2
180kD
130kD
ER alpha
100kD
70kD
55kD
FOXM1
130kD
100kD
PARP
130kD
100kD
cleaved
Caspase-7
25kD
15kD
actin
70kD
55kD
40kD
35kD

Figure 4A

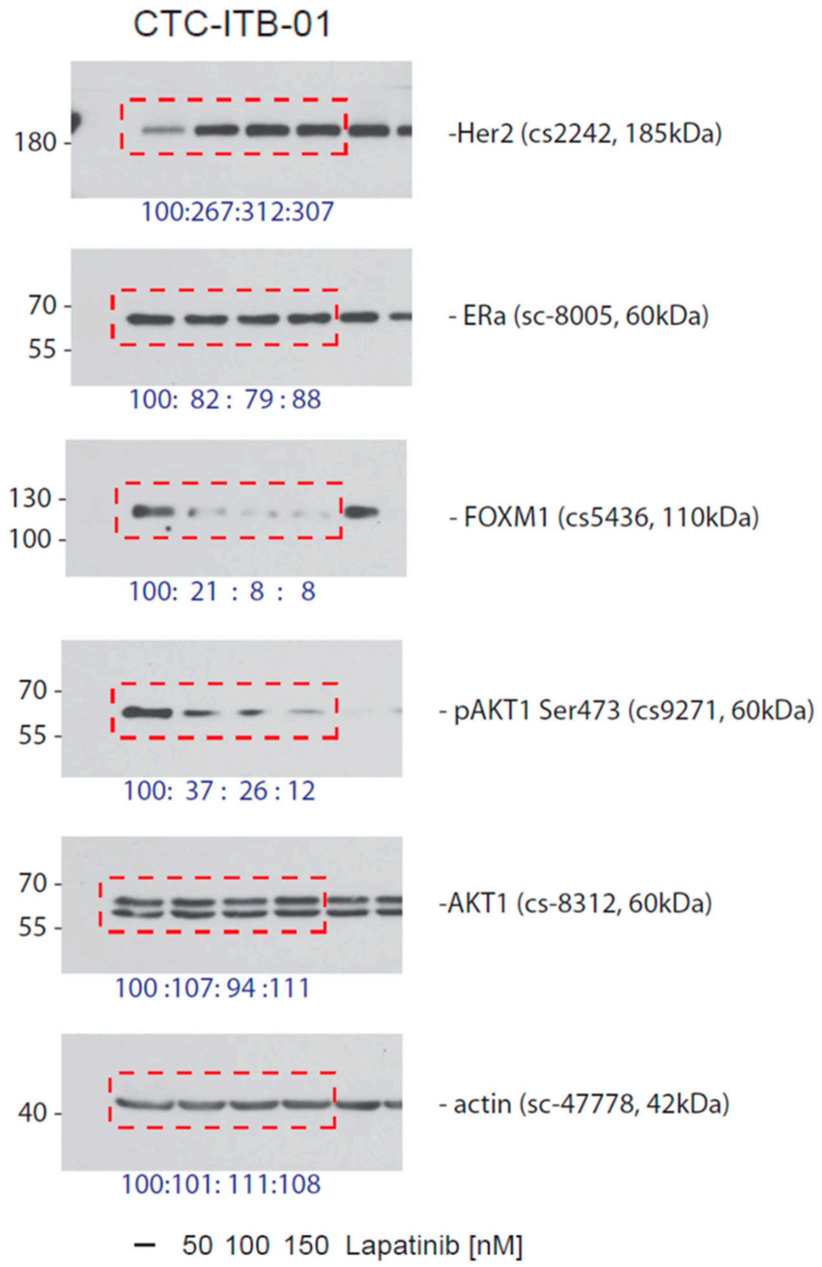
CTC-ITB-01
180 -
-Her2 (cs2242, 185kDa)
100:267:312:307
70 -
55 -
- ERa (sc-8005, 60kDa)
100: 82 : 79 : 88
130 -
100 -
- FOXM1 (cs5436, 110kDa)
100: 21 : 8 : 8
70 -
55 -
- pAKT1 Ser473 (cs9271, 60kDa)
100: 37 : 26 : 12
70 -
55 -
-AKT1 (cs-8312, 60kDa)
100 :107: 94 :111
40 -
- actin (sc-47778, 42kDa)
100:101: 111:108
— 50 100 150 Lapatinib [nM]

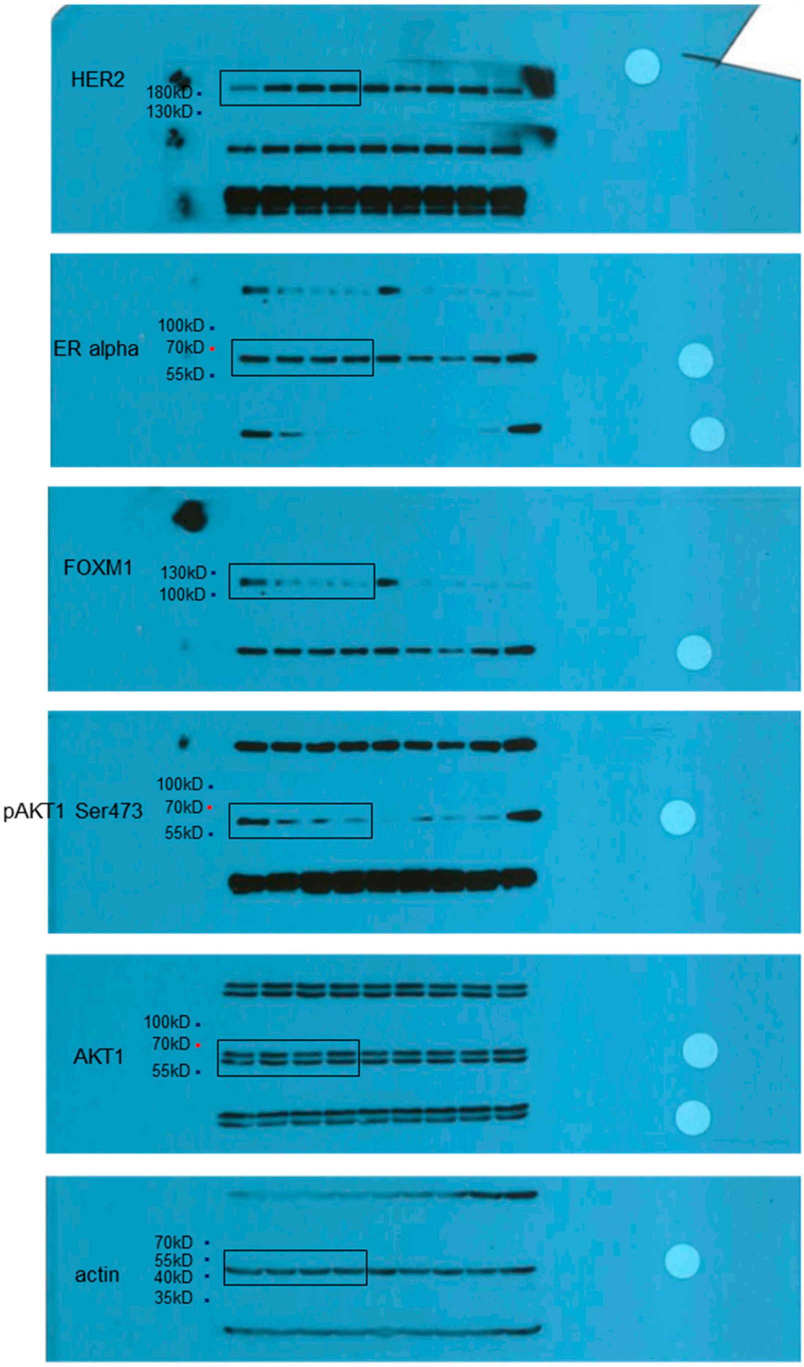
HER2
180kD
130kD
ER alpha
100kD
70kD
55kD
FOXM1
130kD
100kD
pAKT1 Ser473
100kD
70kD
55kD
AKT1
100kD
70kD
55kD
actin
70kD
55kD
40kD
35kD

Figure 4B

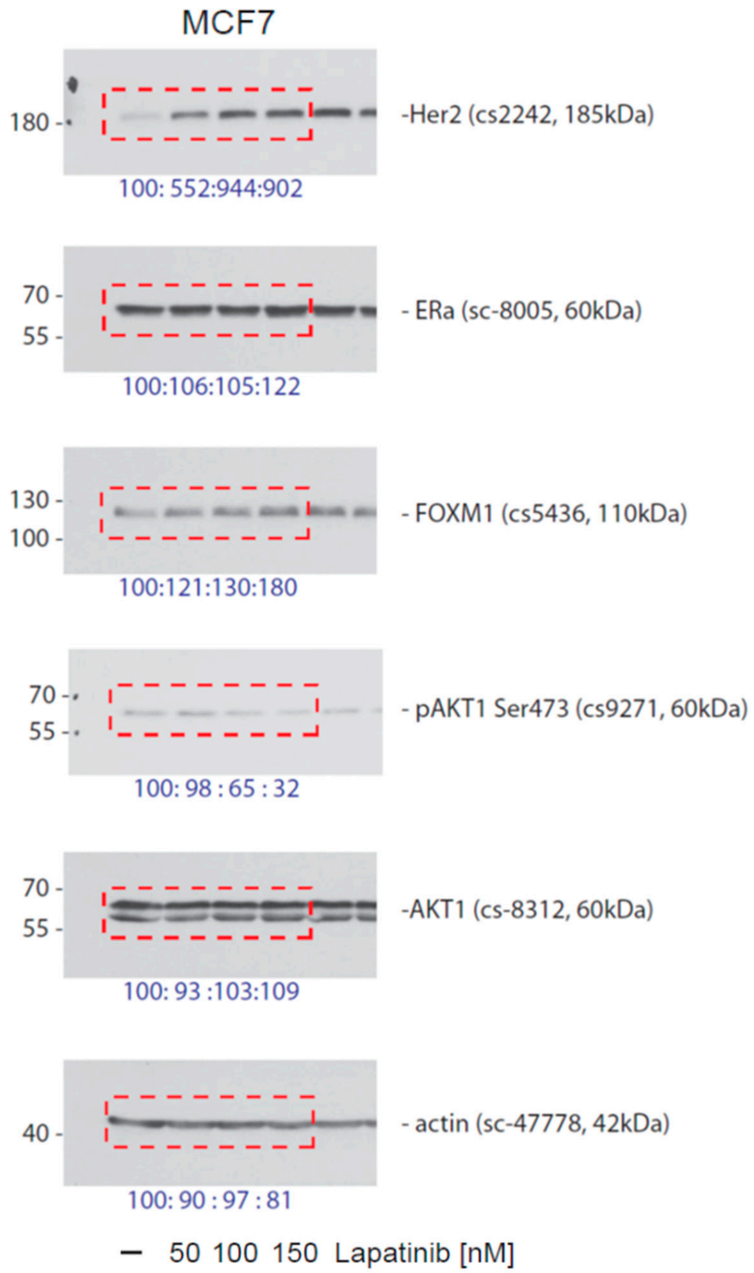
MCF7
180 -
-Her2 (cs2242, 185kDa)
100: 552:944:902
70 -
55 -
- ERa (sc-8005, 60kDa)
100:106:105:122
130 -
100 -
- FOXM1 (cs5436, 110kDa)
100:121:130:180
70 -
55 -
- pAKT1 Ser473 (cs9271, 60kDa)
100: 98 : 65 : 32
70 -
55 -
-AKT1 (cs-8312, 60kDa)
100: 93 :103:109
40 -
- actin (sc-47778, 42kDa)
100: 90 : 97 : 81
— 50 100 150 Lapatinib [nM]

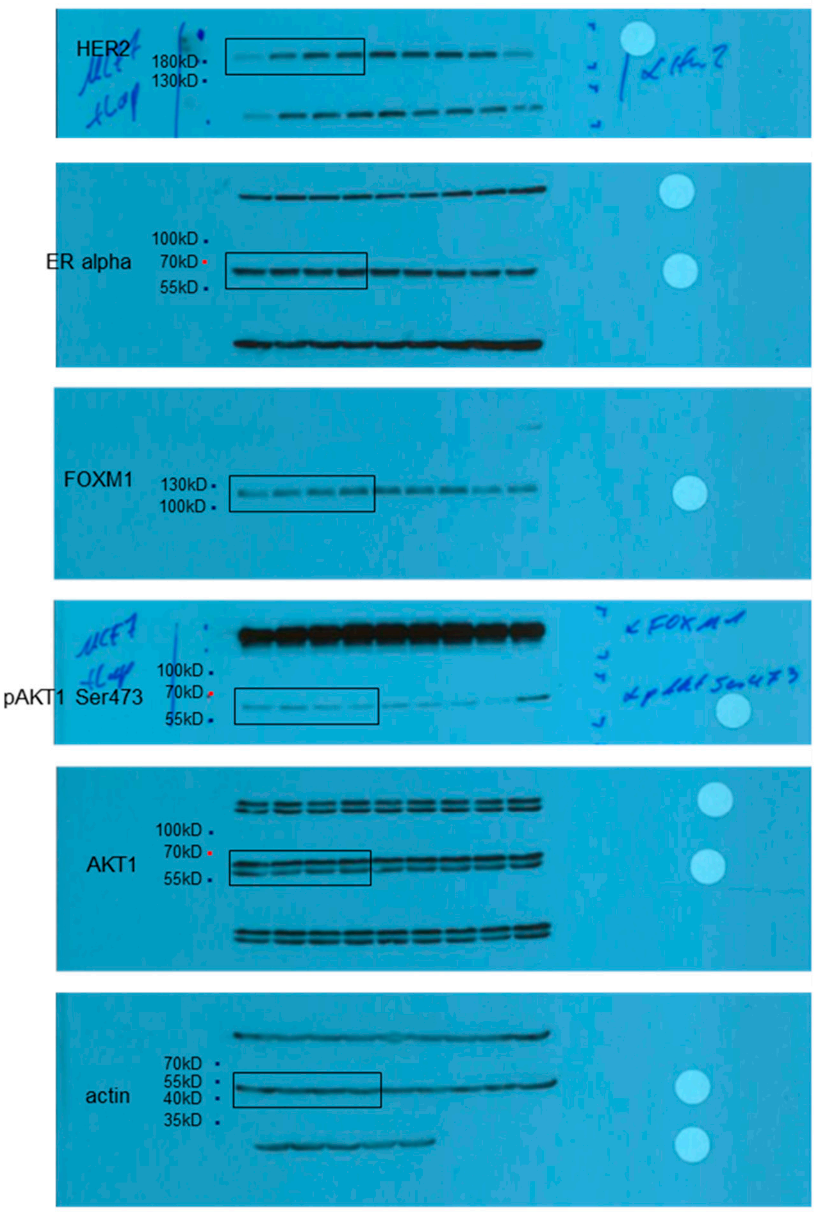
HER2
180kD
130kD
ER alpha
100kD
70kD
55kD
FOXM1
130kD
100kD
pAKT1 Ser473
100kD
70kD
55kD
AKT1
100kD
70kD
55kD
actin
70kD
55kD
40kD
35kD

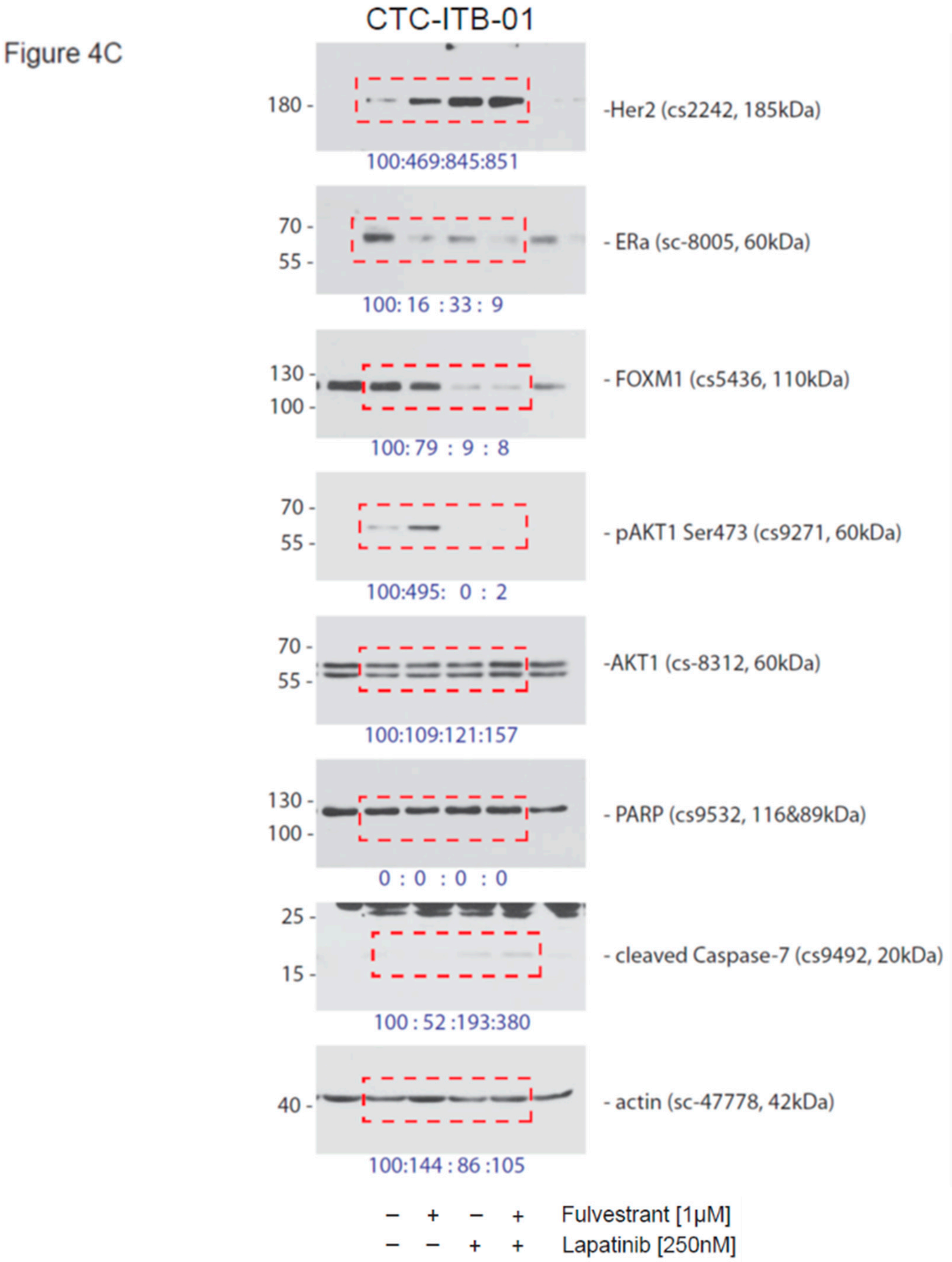
Figure 4C
CTC-ITB-01
180 -
-Her2 (cs2242, 185kDa)
100:469:845:851
70 -
55 -
- ERa (sc-8005, 60kDa)
100: 16 : 33 : 9
130 -
100 -
- FOXM1 (cs5436, 110kDa)
100: 79 : 9 : 8
70 -
55 -
- pAKT1 Ser473 (cs9271, 60kDa)
100:495: 0 : 2
70 -
55 -
-AKT1 (cs-8312, 60kDa)
100:109:121:157
130 -
100 -
- PARP (cs9532, 116&89kDa)
0 : 0 : 0 : 0
25 -
15 -
- cleaved Caspase-7 (cs9492, 20kDa)
100 : 52 :193:380
40 -
- actin (sc-47778, 42kDa)
100:144 : 86 :105
– + – + Fulvestrant [1μM]
– – + + Lapatinib [250nM]

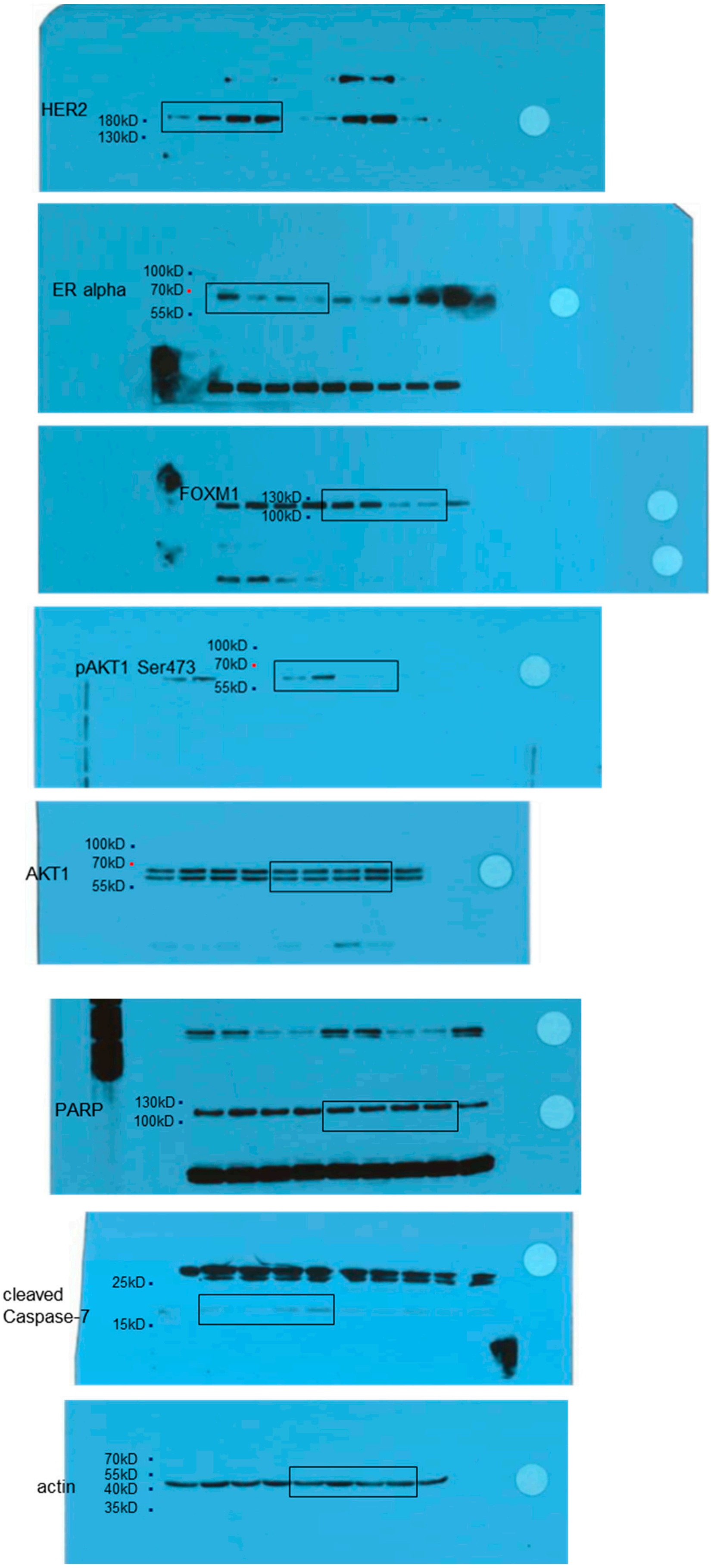
HER2
180kD
130kD
ER alpha
100kD
70kD
55kD
FOXM1
130kD
100kD
pAKT1 Ser473
100kD
70kD
55kD
AKT1
100kD
70kD
55kD
PARP
130kD
100kD
cleaved
Caspase-7
25kD
15kD
actin
70kD
55kD
40kD
35kD

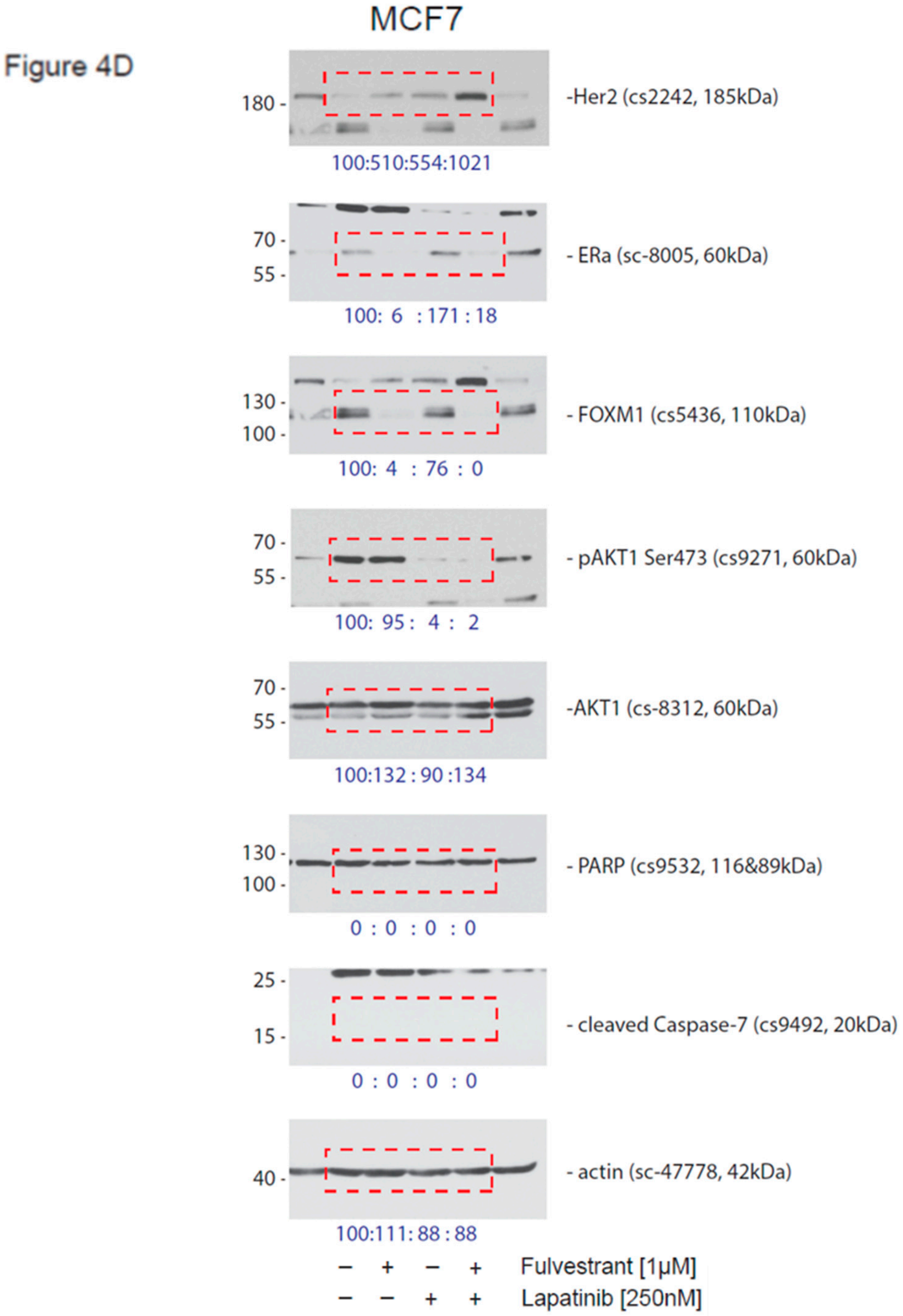
Figure 4D
MCF7
180 -
-Her2 (cs2242, 185kDa)
100:510:554:1021
70 -
55 -
- ERa (sc-8005, 60kDa)
100: 6 : 171 : 18
130 -
100 -
- FOXM1 (cs5436, 110kDa)
100: 4 : 76 : 0
70 -
55 -
- pAKT1 Ser473 (cs9271, 60kDa)
100: 95 : 4 : 2
70 -
55 -
-AKT1 (cs-8312, 60kDa)
100:132 : 90 :134
130 -
100 -
- PARP (cs9532, 116&89kDa)
0 : 0 : 0 : 0
25 -
15 -
- cleaved Caspase-7 (cs9492, 20kDa)
0 : 0 : 0 : 0
40 -
- actin (sc-47778, 42kDa)
100:111: 88 : 88
− + − + Fulvestrant [1μM]
− − + + Lapatinib [250nM]

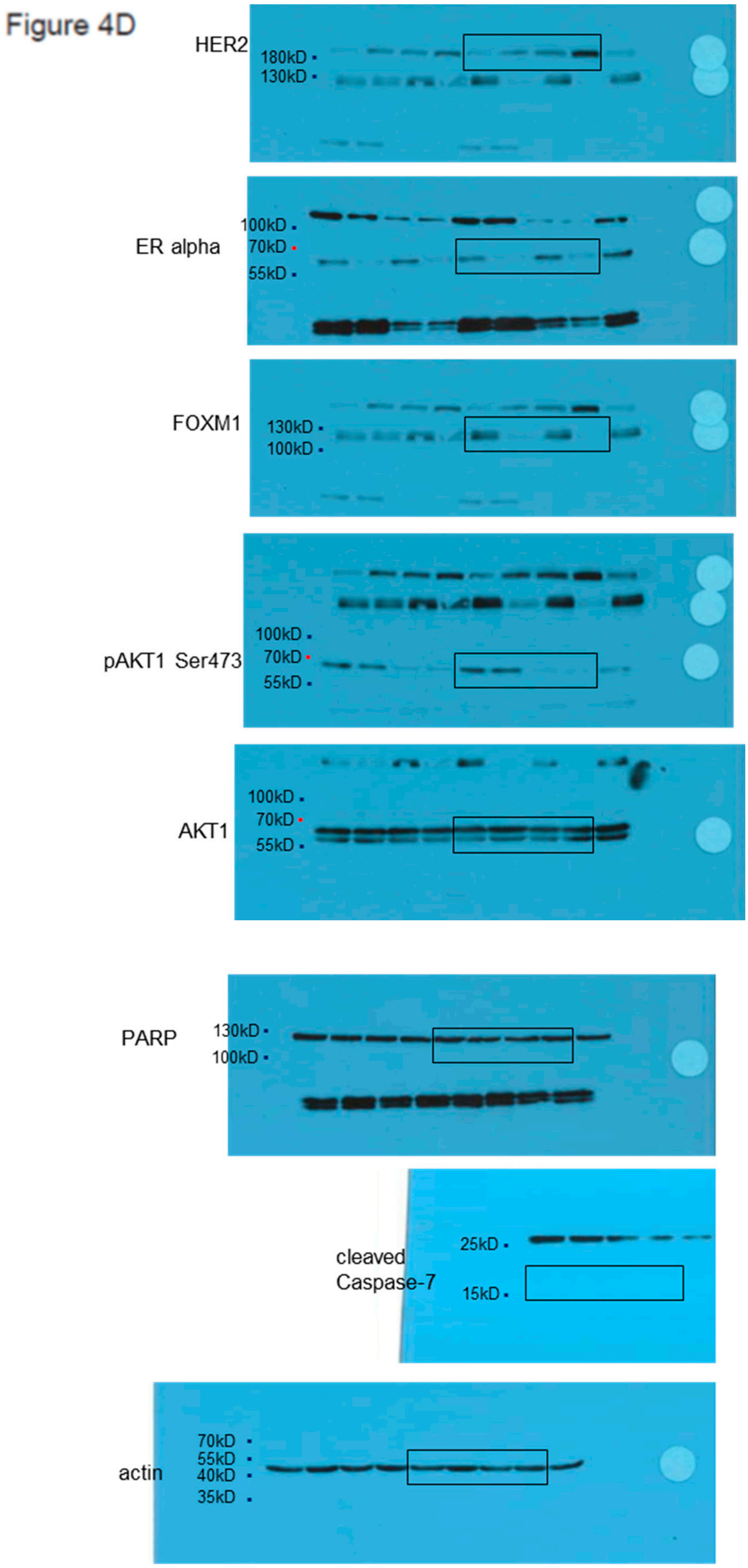
Figure 4D
HER2
180kD
130kD
ER alpha
100kD
70kD
55kD
FOXM1
130kD
100kD
pAKT1 Ser473
100kD
70kD
55kD
AKT1
100kD
70kD
55kD
PARP
130kD
100kD
cleaved Caspase-7
25kD
15kD
actin
70kD
55kD
40kD
35kD

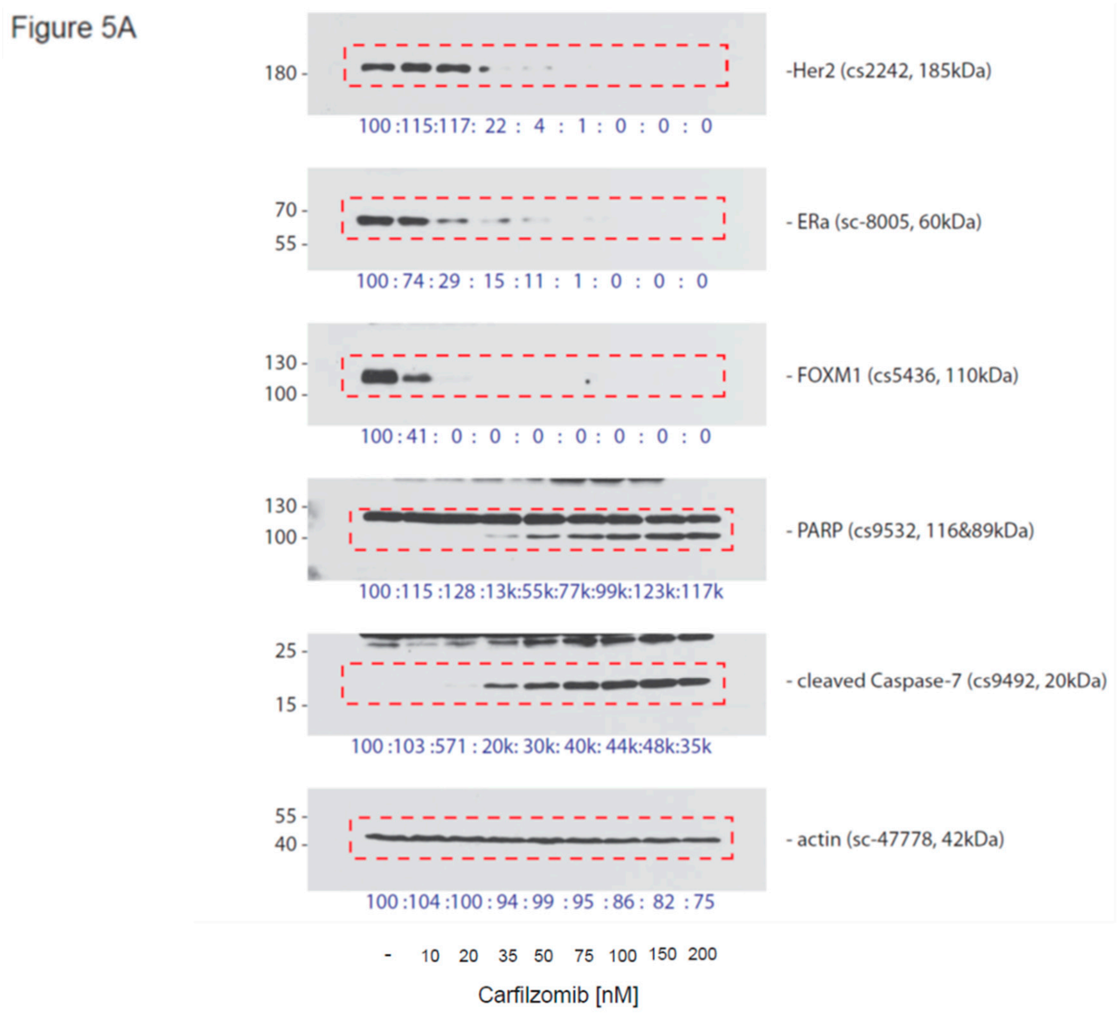

Figure 5A
180 -
-Her2 (cs2242, 185kDa)
100 :115:117: 22 : 4 : 1 : 0 : 0 : 0
70 -
55 -
- ERa (sc-8005, 60kDa)
100 : 74 : 29 : 15 : 11 : 1 : 0 : 0 : 0
130 -
100 -
- FOXM1 (cs5436, 110kDa)
100 : 41 : 0 : 0 : 0 : 0 : 0 : 0 : 0
130 -
100 -
- PARP (cs9532, 116&89kDa)
100 :115 :128 :13k:55k:77k:99k:123k:117k
25 -
15 -
- cleaved Caspase-7 (cs9492, 20kDa)
100 :103 :571 : 20k: 30k: 40k: 44k:48k:35k
55 -
40 -
- actin (sc-47778, 42kDa)
100 :104 :100 : 94 : 99 : 95 : 86 : 82 : 75
- 10 20 35 50 75 100 150 200
Carfilzomib [nM]

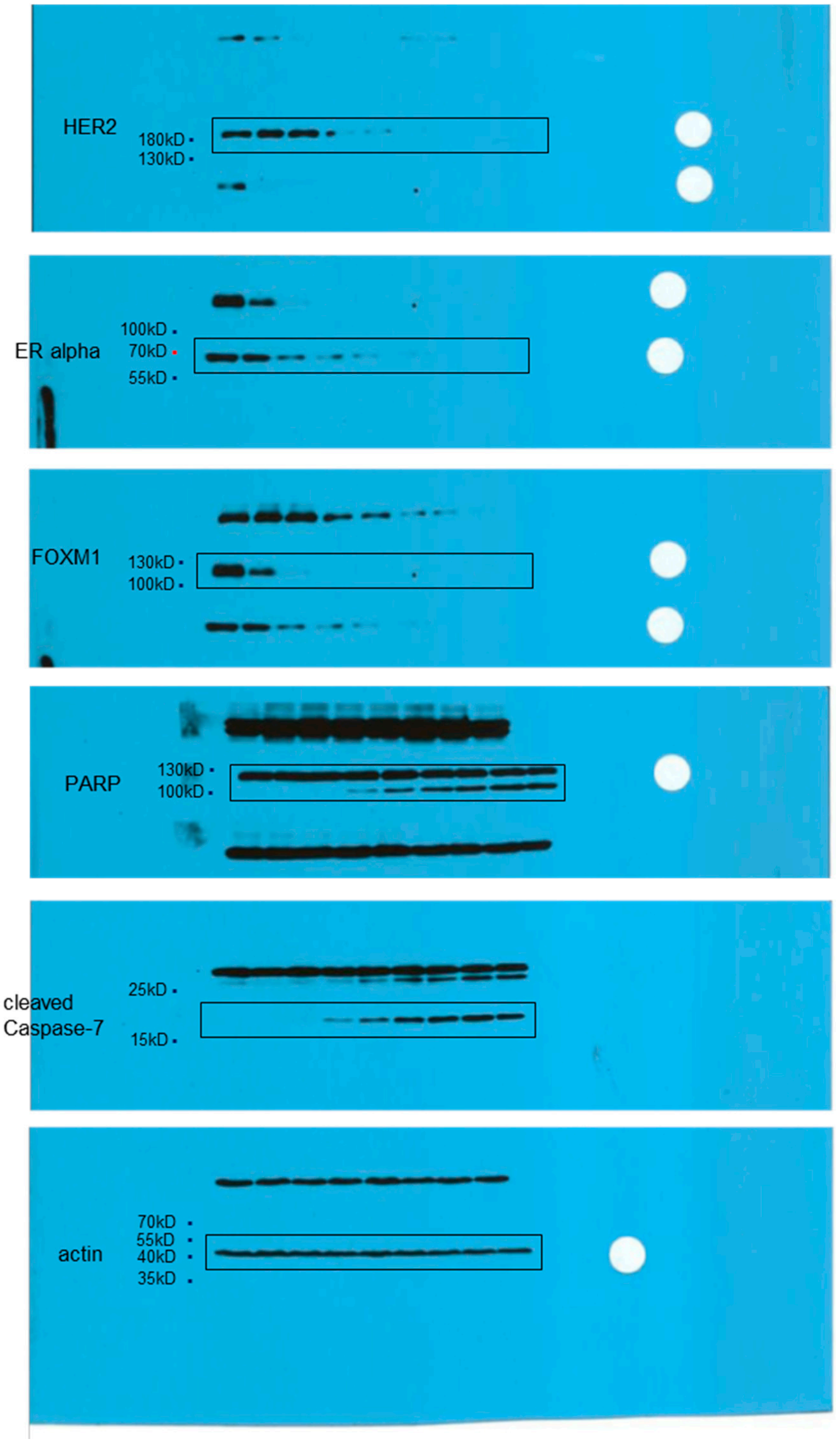
HER2
180kD
130kD
ER alpha
100kD
70kD
55kD
FOXM1
130kD
100kD
PARP
130kD
100kD
cleaved
Caspase-7
25kD
15kD
actin
70kD
55kD
40kD
35kD

Figure 6A

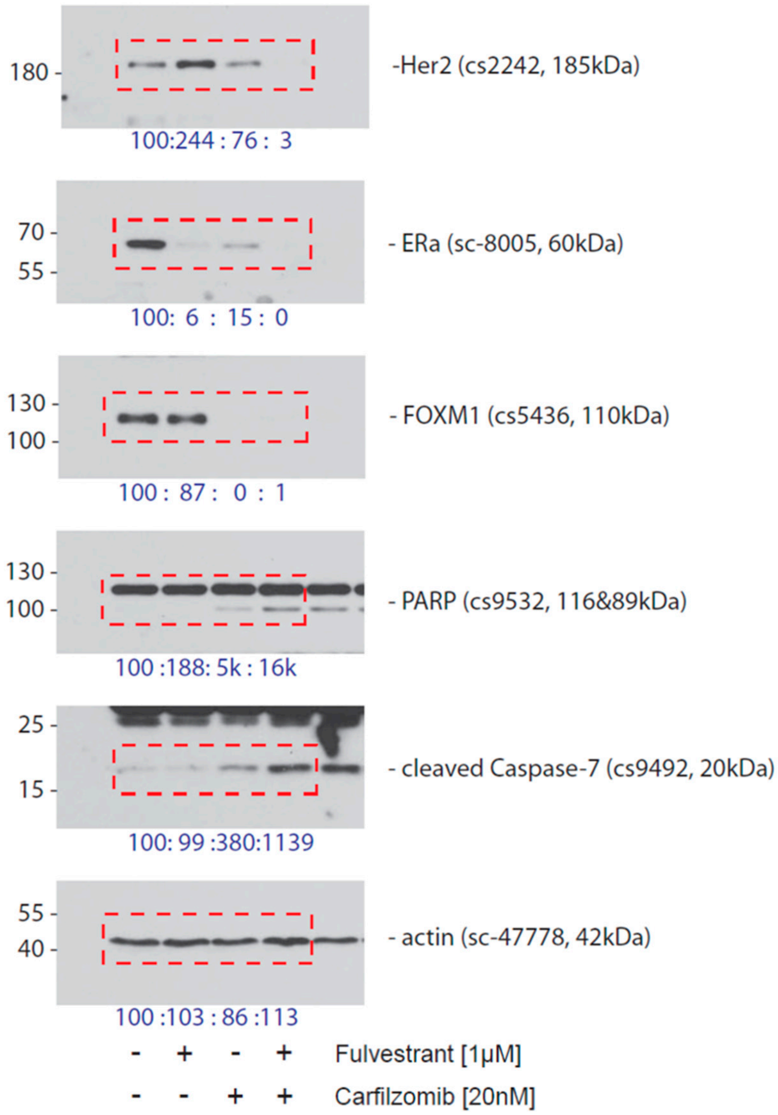

180 -
-Her2 (cs2242, 185kDa)
100:244 : 76 : 3
70 -
55 -
- ERa (sc-8005, 60kDa)
100: 6 : 15 : 0
130 -
100 -
- FOXM1 (cs5436, 110kDa)
100 : 87 : 0 : 1
130 -
100 -
- PARP (cs9532, 116&89kDa)
100 :188: 5k : 16k
25 -
15 -
- cleaved Caspase-7 (cs9492, 20kDa)
100: 99 :380:1139
55 -
40 -
- actin (sc-47778, 42kDa)
100 :103 : 86 :113
- + - + Fulvestrant [1μM]
- - + + Carfilzomib [20nM]

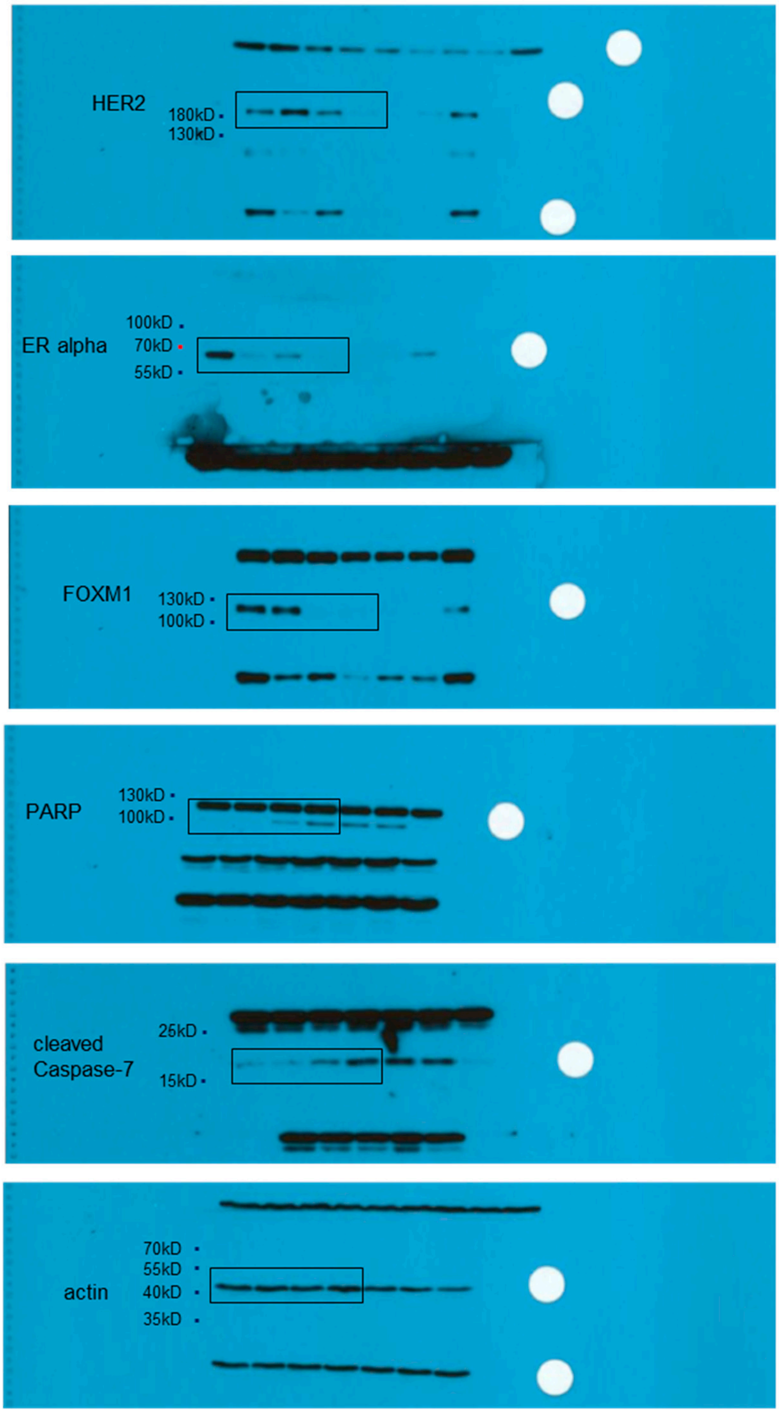
HER2
180kD
130kD
ER alpha
100kD
70kD
55kD
FOXM1
130kD
100kD
PARP
130kD
100kD
cleaved
Caspase-7
25kD
15kD
actin
70kD
55kD
40kD
35kD

Figure 6D

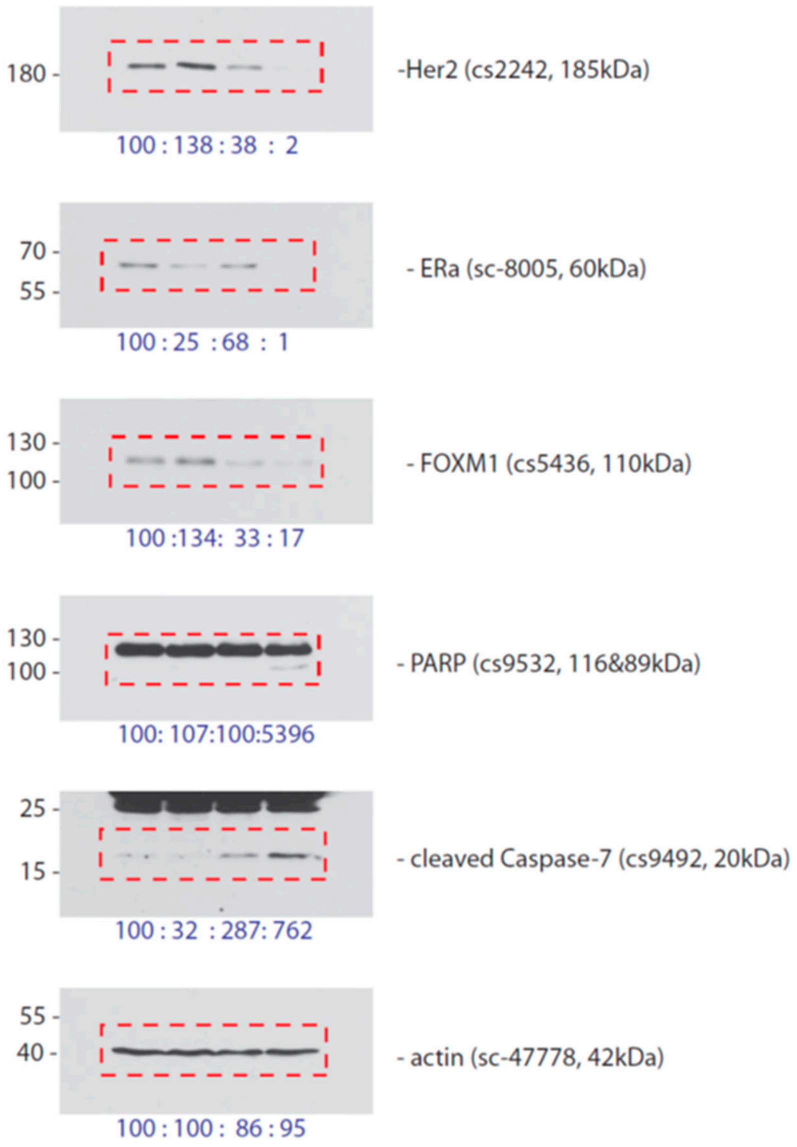
180 -
-Her2 (cs2242, 185kDa)
100 : 138 : 38 : 2
70 -
55 -
- ERa (sc-8005, 60kDa)
100 : 25 : 68 : 1
130 -
100 -
- FOXM1 (cs5436, 110kDa)
100 :134: 33 : 17
130 -
100 -
- PARP (cs9532, 116&89kDa)
100: 107:100:5396
25 -
15 -
- cleaved Caspase-7 (cs9492, 20kDa)
100 : 32 : 287: 762
55 -
40 -
- actin (sc-47778, 42kDa)
100 : 100 : 86 : 95

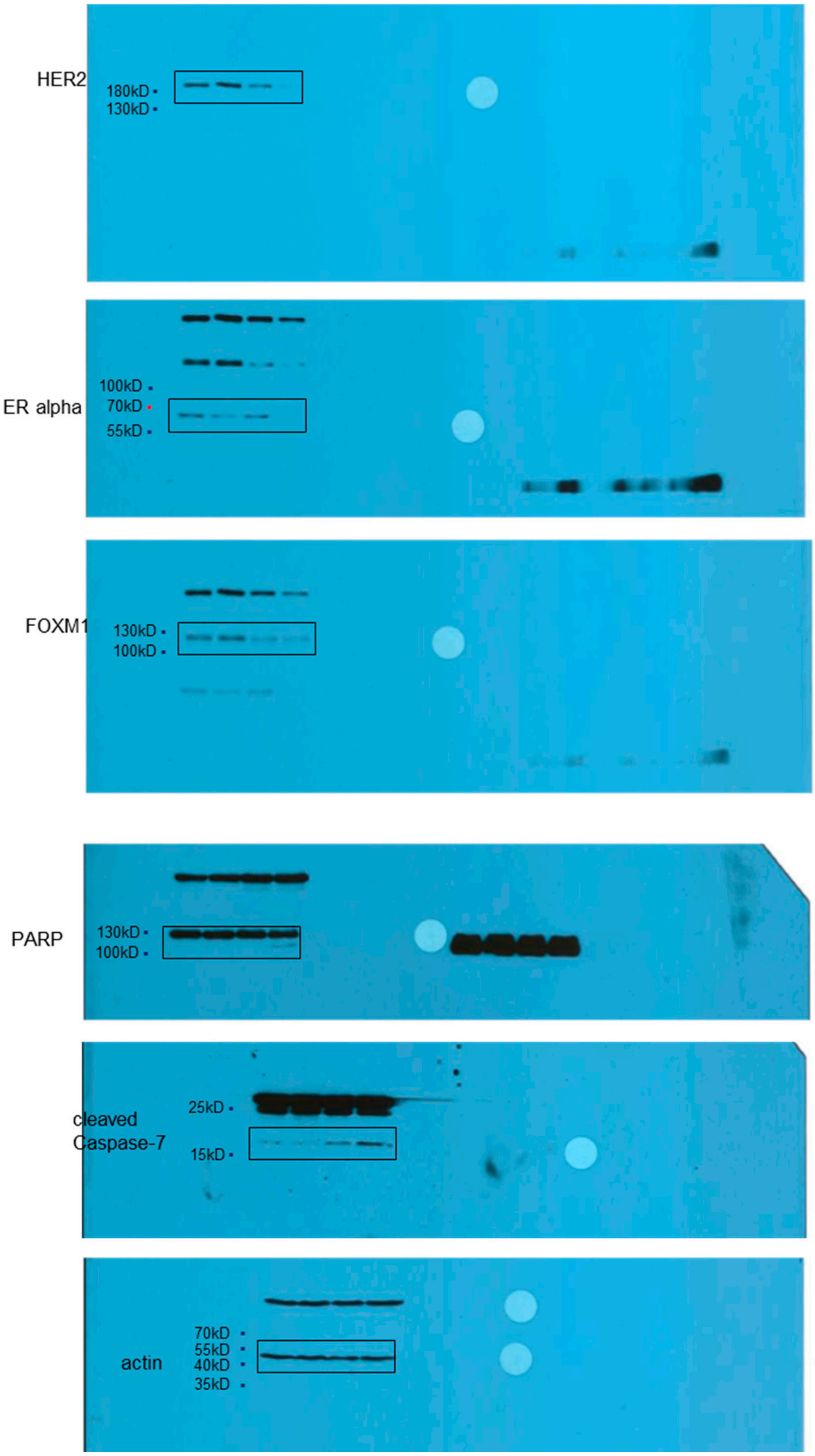
HER2
180kD
130kD
ER alpha
100kD
70kD
55kD
FOXM1
130kD
100kD
PARP
130kD
100kD
cleaved
Caspase-7
25kD
15kD
actin
70kD
55kD
40kD
35kD

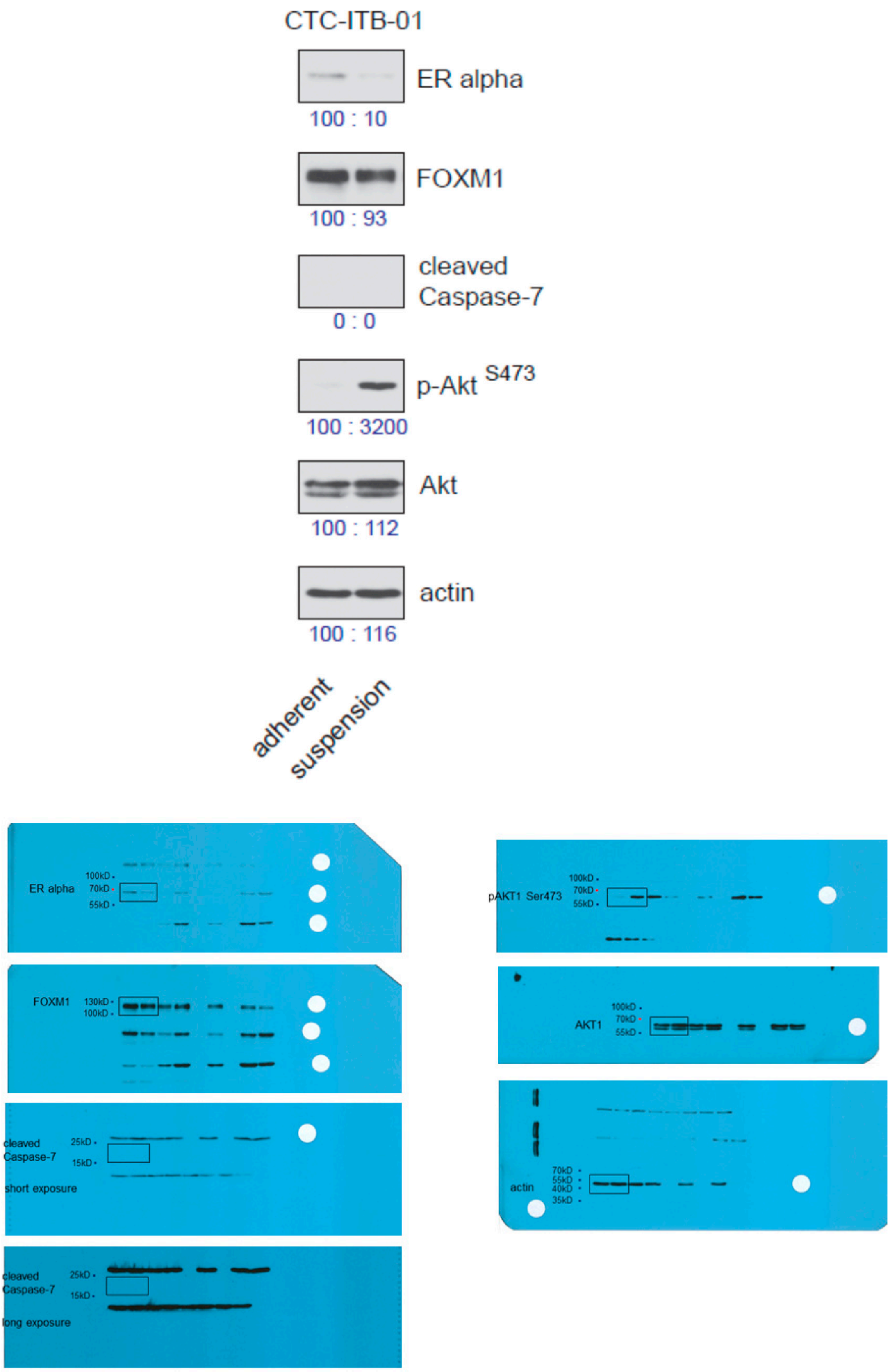
CTC-ITB-01
ER alpha
100 : 10
FOXM1
100 : 93
cleaved Caspase-7
0 : 0
p-Akt S473
100 : 3200
Akt
100 : 112
actin
100 : 116
adherent
suspension
ER alpha
100kD
70kD
55kD
FOXM1
130kD
100kD
cleaved Caspase-7
25kD
15kD
short exposure
cleaved Caspase-7
25kD
15kD
long exposure
pAKT1 Ser473
100kD
70kD
55kD
AKT1
100kD
70kD
55kD
actin
70kD
55kD
40kD
35kD

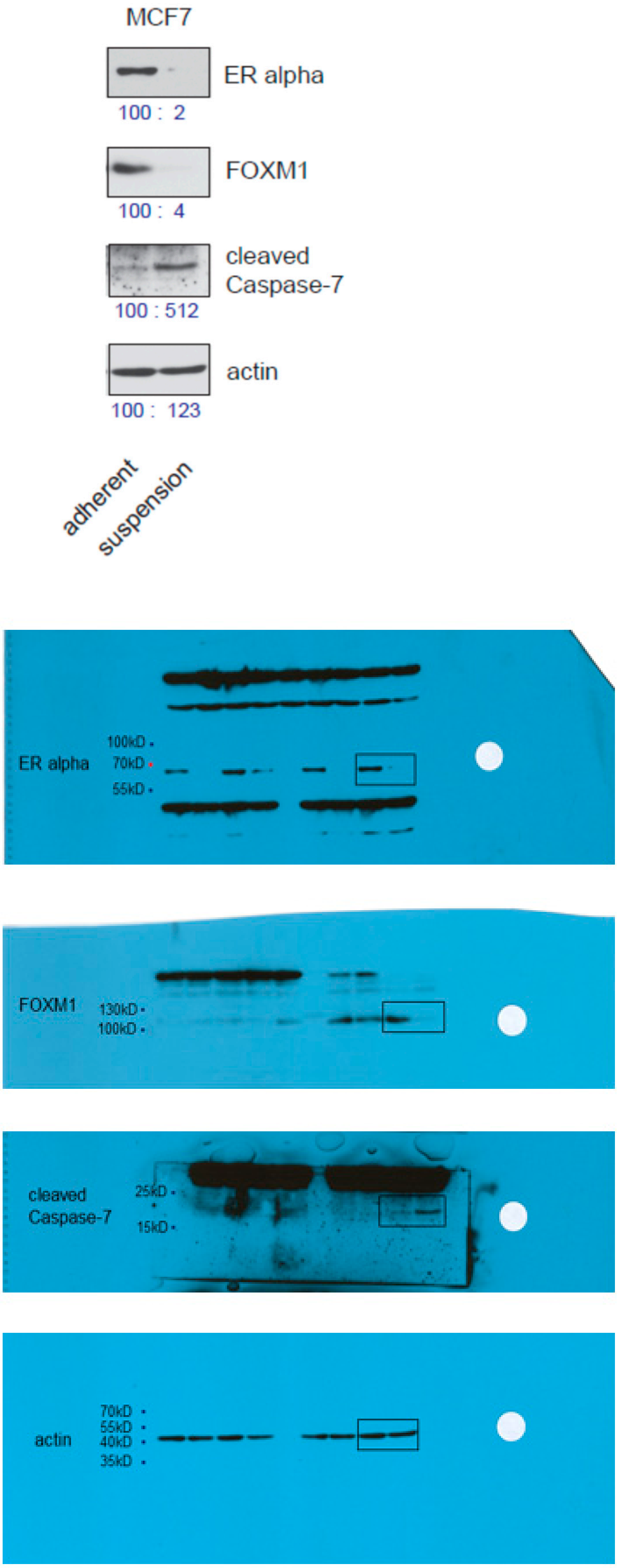

Figure S7. Original Uncropped Western Blot Images.
